# Symbolic regression for strength prediction of eccentrically loaded concrete-filled steel tubular columns

**DOI:** 10.1038/s41598-025-85371-x

**Published:** 2025-01-24

**Authors:** Khaled Megahed

**Affiliations:** Department of Structural Engineering, Mansoura University, PO BOX 35516, Mansoura, Egypt

**Keywords:** Concrete-filled-steel tubular columns, Design code standards, Machine learning, Symbolic regression, CCFST, RCFST, CatBoost model, Civil engineering, Scientific data, Statistics

## Abstract

Concrete-filled steel tube (CFST) columns are widely employed in high-rise buildings, long-span bridges, and seismic-resistant structures due to their superior load-bearing capacity, structural efficiency, and resilience under extreme loading conditions. This study uses symbolic regression with structural design code provisions to predict the eccentric strength of concrete filled-steel tubular columns with circular shape (CCFST) and rectangular shape (RCFST). Previous studies have used two distinct approaches for estimating eccentric strength: explainable models based on theoretical derivations and black-box models derived from machine learning (ML) methods. This study proposes a hybrid model derived from the design code standards, with performance enhanced by the symbolic regression technique. This model is based on a comprehensive experimental database of 464 tests for CCFST columns and 313 tests for RCFST columns under eccentric loading from various research papers. The developed code-based symbolic regression (C-SR) displays both robust and interpretable, demonstrating high prediction accuracy with mean values of the prediction-to-actual ratios of 1.006 and 0.997 and coefficient of variation (CoV) values of 0.117 and 0.098 for CCFSTs and RCFSTs, respectively, while providing explainable mathematical expressions that align with the mechanical principles of code provisions. The developed C-SR model is benchmarked against EC4 and AISC360 standards and evaluated against the various ML techniques, demonstrating acceptable performance. The results highlight the C-SR model’s effectiveness in providing reliable predictions and valuable insights for practical engineering applications.

**Supplementary Information:**

The online version contains supplementary material available at 10.1038/s41598-025-85371-x.

## Introduction

Concrete-filled steel tube (CFST) members are composite structural systems comprising hollow steel tubes filled with concrete, offering significant advantages over both steel and traditional reinforced concrete columns. These advantages include enhanced structural efficiency, increased load-bearing capacity, and improved ductility^1^. The steel tube serves to confine the concrete core, effectively delaying or preventing lateral expansion and failure, while the concrete core limits the inward local buckling of the steel tube^1,2^. Additionally, CFST columns exhibit excellent resistance to fire and seismic loads while also acting as permanent formwork, which helps fasten construction processes.

CFST columns are available in various configurations based on loading conditions and geometric design, including conventional CFST columns and concrete-filled double-skin steel tubular columns (CFDSTs), which incorporate an extra inner hollow steel tube. The cross-sectional shapes of these columns vary and may include circular, rectangular, octagonal, hexagonal, or elliptical profiles^3–6^. Circular CFST columns are particularly selected for their ability to provide uniform confinement to the concrete core, resulting in higher load capacity and greater ductility. Recently, Hamoda et al.^7^ explored various configurations for strengthening columns using external stainless steel plates, resulting in an increase in load capacity by 26–112% and absorbed energy by 34–190%. Ahmed et al.^8^ developed a new confinement model for rectangular CFST columns based on test results, accounting for the confinement induced by the embedded steel section, and validated its accuracy using experimental data. On the other hand, rectangular CFST columns are often chosen for their simplicity in construction and ease of connection assembly.

Experimental studies are frequently conducted to understand the behavior of CFST columns. However, these investigations are often constrained by limited parameter ranges and can be both expensive and time-consuming. In contrast, machine learning (ML) techniques have emerged as valuable tools to complement experimental work, demonstrating considerable success in predicting the behavior of structural elements. Researchers have employed ML algorithms such as Gaussian Process Regression (GPR)^9,10^, gene expression programming (GEP)^11^, and artificial neural network (ANN)^12–15^ to develop empirical formulas and statistical models for predicting material properties like strength and elastic modulus, as well as the performance of structural members.

ML methods are particularly effective for predicting the ultimate capacity of CFST columns. For instance, Ahmadi et al.^12,13^ utilized ANN models to predict the ultimate strength of short CFST columns. Tran et al.^14^ assembled a database of 300 samples subjected to uniaxial loading to train ML models for calculating the axial strength of square CFST columns. Zarringol et al.^15^ gathered four databases containing a total of 3091 CFST columns, covering rectangular and circular columns with and without eccentricity. They developed four distinct ANN models for each category’s axial capacity, integrating strength reduction factors to enhance their applicability in practical design. Asteris et al.^16^ developed an ANN model to predict the ultimate strength of RCFST columns under axial and eccentric loading. The developed model was compared against available design codes, displaying significant accuracy and stable numerical behavior. Nguyen and Kim^17^ employed an ANN optimized through particle swarm optimization (PSO) to predict the capacity of RCFST columns under axial and eccentric loading. Hou and Zhou^18^ utilized various ML techniques, including ANN, GPR, genetic algorithm, radial basis function neural network, and multiple linear regression (MLR) models, to predict the axial capacity of both short and long circular CFST columns.

Additionally, GEP, genetic algorithms (GAs)^11^, and symbolic regression (SR)^19,20^ have proven helpful in deriving empirical formulas for the ultimate strength of CFST columns. Javed et al.^21^ applied GEP to predict the load-bearing strength of circular CFST long columns, while Jiang et al.^22^ compared GEP predictions with finite element analysis results for circular CFST columns. Naser et al.^23^ utilized GA and GEP to develop models predicting the axial ultimate load of rectangular and circular CFST columns. Ipek and Güneyisi^24^ implemented GEP and ANN for strength prediction of CFST elliptical columns under eccentric loading. Megahed et al.^19^ implemented SR model with various ML models for predicting the axial strength of stub columns with different cross-section shapes. Table 1 provides an overview of previous studies utilizing GEP and GA models for predicting CFST column strength.

**Table 1:** Summary of previous ML models in predicting the strength of axially loaded stub CFST columns.

Reference	Category (number) type [Split ratio%]	Input (output)	Models: statistical criteria
Ahmadi^12,13^	Axial (272) Circular [80:20]	*L*,* D*,* t*,* f*_*y*_, *f*_*c*_*’*,*E*_*s*_ (*P*_*u*_)	Regression model: R^2^ = 0.926, MAPE% = 13.2, with expression $\:{P}_{u}=p\left(D\right)p\left({f}_{y}\right)p\left({f}_{c}^{{\prime\:}}\right)p\left(t\right)p\left(L\right)$ where $\:p\left(x\right)$ is a third-degree to six-degree polynomial function of x.
Tran^45^	Axial (258) Circular [85:15]	*L*,* D*,* t*,* f*_*y*_, *f*_*c*_*’* (*P*_*u*_)	Regression model: with expression $\:{P}_{u}=p\left(D\right)p\left({f}_{y}\right)p\left({f}_{c}^{{\prime\:}}\right)p\left(t\right)p\left(L\right)$ where $\:p\left(x\right)$ is a polynomial function of x. µ = 1.04, CoV = 0.24, MAPE = 0.18, MAE=323.52, RMSE=565, R^2^ = 0.95
Tran^14^	Axial (300) Square [85:15]	*L*,* H*,* t*,* f*_*y*_, *f*_*c*_*’* (*P*_*u*_)	Regression model: with expression $\:{P}_{u}=p\left(H\right)p\left({f}_{y}\right)p\left({f}_{c}^{{\prime\:}}\right)p\left(t\right)p\left(L\right)$ where $\:p\left(x\right)$ is a polynomial function of x. MAPE = 0.22, MAE=559.7, RMSE = 789.8, R2 = 0.98, µ = 1.18, CoV = 0.25
Guneyisi^46^	Axial (314) Circular [75:25]	*L*,* D*,* t*,* f*_*y*_, *f*_*c*_*’* (*P*_*u*_)	$\:\text{G}\text{E}\text{P}:\:\text{w}\text{i}\text{t}\text{h}\:{P}_{u}={P}_{1}{P}_{2}{P}_{3}{P}_{4}{P}_{5}{P}_{6}\:\text{w}\text{h}\text{e}\text{r}\text{e}\text{*}\:{P}_{1}=\text{sin}\left[{sin}\left(\frac{24.4\left(t-D\right)}{{f}_{c}^{{\prime\:}}}+\left({f}_{c}^{{\prime\:}}-24.4+{10}^{24}\right)\right)\right]-{f}_{y}$ MAPE% = 7.49, RMSE = 228
Ipek^37^	Axial (103) Double skin [75:25]	*L*,* D t*,* D*_*i*_, *t*_*i*_, *f*_*y*_, *f*_*c*_*’*,* f*_*yi*_ (*P*_*u*_)	$\:\text{G}\text{E}\text{P}:\:\text{w}\text{i}\text{t}\text{h}\:{P}_{u}={P}_{1}{+P}_{2}+{P}_{3}+{P}_{4}+{P}_{5}+{P}_{6}\:\text{w}\text{h}\text{e}\text{r}\text{e}\text{*}\:{P}_{1}=Dt-D\text{cos}\left(t-{D}_{i}-\frac{{f}_{c}^{{\prime\:}}}{47.4}\right)$ MAPE% = 6.43, RMSE = 85.7, R^2^ = 0.987, a20-index = 0.884, µ = 1.003, CoV = 0.084
Javed^21^	Axial (227) circular [78:22]	*L*,* D*,* t*,* f*_*y*_, *f*_*c*_*’* (*P*_*u*_)	GEP: with $\:{P}_{u}=D\left(3t-1\right)-{t}^{2}-137.67t-\left(4t+1\right)\frac{L}{D}+\frac{{f}_{y}}{t}+6.72{f}_{c}^{{\prime\:}}+{\left({f}_{y}-L\right)}^{\frac{1}{3}}-46.61$ RMSE = 258, R^2^ = 0.98, MAE = 138.7, µ = 1.2, CoV = 0.1
Jiang^22^	Axial (22) Circular	*L*,* D*,* t*,* f*_*y*_, *f*_*c*_*’* (*P*_*u*_)	GEP: $P_{u} = 2D\sqrt {f_{c}^{'} } + f_{y} \left( {\sqrt D - \left( {6.219 - t} \right)} \right) + \left[ {8.078f_{y} + 0.626L} \right]/\tanh \left( { - 2.831} \right)~$
Naser^23^	Axial circular (1245), rectangular (979) [70:30]	*L*,* D*,* H*,* B*,* t*,* f*_*y*_, *f*_*c*_*’* (*P*_*u*_)	GA^+^: $\:{P}_{u}=0.00439Dt{f}_{y}+0.00072t{D}^{2}+0.00727{f}_{c}^{{\prime\:}}{D}^{2}+1.38\times\:{10}^{-5}DL{f}_{c}^{{\prime\:}}-3.7\times\:{10}^{-7}DtL{f}_{y}$ µ = 1.02, CoV = 0.13, MAE = 202, RMSE = 295 GEP: µ = 1.06, CoV = 0.15, MAE = 238, RMSE = 340
Memarzadeh 2023^47^	Axial circular (646) square (347) [85:15]	*f*_*y*_, *f*_*c*_’,*A*_*c*_,*A*_*s*_, *B/t*,$\:\lambda\:$ (*P*_*u*_)	GEP: (Circular) $\:{P}_{u}={A}_{s}+2{f}_{c}^{{\prime\:}}-4\lambda\:+\sqrt{{f}_{c}^{{\prime\:}}}\left({A}_{c}+\sqrt{3{f}_{c}^{{\prime\:}}-9.596}\right)+\frac{0.169{A}_{s}\left({f}_{y}-2\lambda\:\right)\sqrt{{A}_{c}-11.562}}{D/t}$ µ = 0.98, CoV = 0.22, R^2^ = 0.98, a20-index = 72.14, MAE = 242, RMSE = 384 GEP: (Square) $\:{P}_{u}=3{A}_{c}++9.669{A}_{s}-\frac{B}{t}+{f}_{y}+{A}_{s}{f}_{c}-\frac{\left({f}_{c}^{{\prime\:}}{A}_{s}^{2}+{f}_{c}^{{\prime\:}}\right)}{{A}_{c}}-\frac{22.27\lambda\:\left({f}_{c}^{{\prime\:}}-\lambda\:+188.36\right)}{{f}_{y}}$ µ = 0.99, CoV = 0.23, R^2^ = 0.98, a20-index = 0.70, MAE = 324, RMSE = 464
Ipek^24^	Eccentric Elliptical (44)	*L*,* a*,* b*,* t*,* f*_*y*_, *f*_*c*_*’*,* e*_*y*_, *e*_*z*_ (*P*_*u*_)	$\:\text{G}\text{E}\text{P}:{P}_{u}={P}_{1}{+P}_{2}+{P}_{3}+{P}_{4}+{P}_{5}+{P}_{6}+{P}_{7}+{P}_{8}\:\text{w}\text{h}\text{e}\text{r}\text{e}\:{P}_{1}={f}_{c}^{{\prime\:}}ta{n}^{-1}\left({f}_{c}^{{\prime\:}}\right)ta{n}^{-1}\left({e}_{y}-{e}_{z}+\frac{L-{e}_{z}}{t}\right)$ $\:{P}_{2}=\sqrt{{e}^{t+\frac{{f}_{st}}{2a{e}^{t}-tL-L}}},\:{P}_{3}=2b-\sqrt{3{f}_{st}+2a{e}_{y}+\left(L+{f}_{st}\right)\sqrt{L}},\:{P}_{4}=6a+2b-\text{ln}\left({e}^{{e}_{Z}}-\rho\:+{f}_{y}\right),\:$ $\:{P}_{5}={e}_{y}-{f}_{c}^{{\prime\:}}\left({e}_{y}-t\right){\text{tan}}^{-1}\left(t\right)\frac{f+{f}_{c}^{{\prime\:}}}{{f}_{st}-2b},\:{P}_{7}=t-\rho\:-{e}_{z}{\text{tan}}^{-1}\left({e}_{Z}{\text{tan}}^{-1}{(f}_{c}^{{\prime\:}})\:+{e}_{z}-2b\right),$ $\:{P}_{6}=\sqrt{t{e}_{z}+{f}_{y}}-{e}_{y}\sqrt{\rho\:}-{f}_{c}^{{\prime\:}}+\sqrt{{f}_{st}},\:{P}_{8}=\sqrt{L-t\left({f}_{c}^{{\prime\:}}-2b{f}_{c}^{{\prime\:}}-3{f}_{y}\right)}-{e}_{y}$ MAPE=7.48, R^2^=0.976
This study	Circular (464) Rectangular (313) [80:20]	Tables 5 and 6	SR: (Circular) µ = 1.006, CoV = 0.117, a20-index = 0.920, MAPE% = 9.223, RMSE = 151.1kN SR: (Rectangular) µ = 0.997, CoV = 0.098, a20-index = 0.946, MAPE% = 7.439, RMSE = 196.5kN

*The remaining parameters* P*_*i*_ have similar expressions to* P*_1_.

+The expression provided is for circular columns. Similar expressions are introduced for rectangular and circular columns using GA and GEP.

Due to the distinct prediction mechanisms between explainable models, such as structural design codes, and black-box models, like data-driven approaches, these methods have traditionally been viewed as independent in resistance prediction^18,19,24^. Design code provisions are valued for their interpretability and are grounded in experimental observations and analytical studies, whereas black-box models are favored for their high predictive accuracy and efficiency. This study applies symbolic regression (SR) to improve the accuracy of design equations from existing code provisions for predicting the strength of CFST columns under eccentric loading. SR is an area of interpretable machine learning based on gene expression programming (GEP), which evolves mathematical expressions to best fit a given dataset. By implementing SR, the standard code design formulas were refined to introduce more precise computational results while maintaining interpretability. This approach serves as a hybrid model or an intermediate solution, effectively bridging the gap between mechanical-based models and black-box approaches, thereby gaining popularity in recent studies^19,25^.

In addition, many studies from previously mentioned literature employ ML models that, while accurate, operate as black-box systems, making them complex and impractical for direct use in design applications. These models often lack interpretability, which limits their adoption in engineering practice. On the other hand, studies that utilize GEP or GA approaches, as provided in Table 1, frequently produce equations that suffer from unit inconsistencies and lack physical meaning, further hindering their practical application. This study addresses these gaps by combining the strengths of interpretable design code provisions with the predictive power of symbolic regression (SR). Unlike traditional ML models, our approach ensures that the refined equations are not only accurate but also interpretable and grounded in physical principles. Additionally, the proposed methodology overcomes the limitations of GEP and GA by generating physically meaningful equations with proper unit consistency, making them both user-friendly and reliable for practical design use. Furthermore, the proposed symbolic regression-based approach offers key advantages over finite element analysis (FEA), including reduced computational demand, simplified modeling preparation, and direct predictions without convergence issues. Its interpretable equations further enhance result accessibility, making it a practical and efficient alternative to FEA and black-box ML models for engineering applications.

The primary objective of this study is to develop a code-based symbolic regression (C-SR) model, which builds on interpretable code provisions to enhance the accuracy of strength predictions for circular (CCFST) and rectangular (RCFST) concrete-filled steel tube columns under eccentric loading. An enforced structure tree for symbolic regression is employed to achieve three key goals: (1) reduce the search space, enhancing the efficiency of the genetic programming process; (2) generate explainable equations consistent with code provisions; and (3) diverge from previous studies that produced uninterpretable SR-generated functions. Here, “interpretability” refers to the model’s ability to produce equations that are computationally feasible and reflect the physical behavior of structures, making them useful and practical for structural engineers. This ensures the resulting models are not only computationally efficient but also maintain interpretability and align with established mechanical principles.

The model was calibrated using a comprehensive database of 464 CCFST and 313 RCFST columns. The input features—geometric configurations, material properties of concrete and steel, and loading eccentricities—are detailed in the subsequent section (see Experimental Database). Multiple evaluation metrics were used to assess the model’s accuracy and generalizability. The predictions from the proposed C-SR model were compared against several machine learning models, including Gaussian Process Regression (GPR), Support Vector Regression (SVR), XGBoost (XGB), CatBoost (CATB), Random Forest (RF), and LightGBM (LGBM), as well as two existing code standards, EC4^26^ and AISC360^27^. This comparison highlights the effectiveness and reliability of the proposed C-SR model, demonstrating its ability to predict the compressive strength of CFST columns under eccentric loading while maintaining interpretability and consistency with the underlying mechanical principles of code provisions.

## Dataset description

**Fig. 1:**
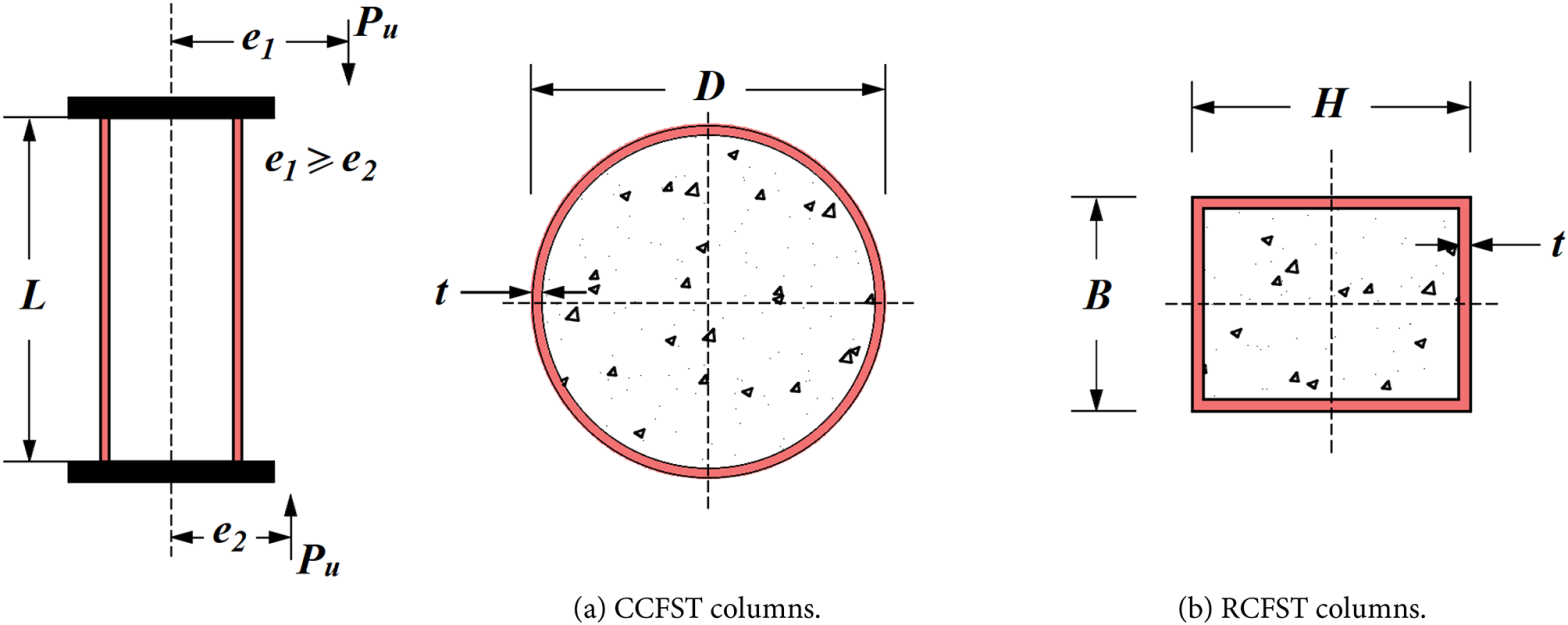
The dimensions of CCFST and RCFST columns.

In this section, a comprehensive experimental dataset comprising 777 column specimens has been carefully selected from studies focused on eccentrically loaded CFST columns. Figure 1 illustrates the loading conditions and geometric configurations of the specimens. All included experiments were conducted on CFST columns subjected to monotonic loading without internal reinforcement, considering only samples uniformly loaded across the entire cross-section. The database is divided into two main parts: (1) Dataset 1 contains 464 observations with seven input parameters for circular CFST (CCFST) columns, and (2) Dataset 2 consists of 313 observations with eight input parameters for rectangular CFST (RCFST) columns.

Table 2 summarizes the key details and the statistical distributions of the specimens, including the outer steel tube diameter (*D* in mm) for circular CFST columns, the outer steel tube width (*B* in mm, measured perpendicular to the eccentricity direction), and the outer steel tube depth (*H* in mm, measured parallel to the eccentricity direction) for rectangular CFST columns. Other parameters include the steel tube thickness (*t* in mm), the compressive strength of the core concrete (*f’*_*c*_ in MPa), the yield strength of the steel tube (*f*_*y*_ in MPa), the top and bottom eccentricities (*e*_*1*_, *e*_*2*_ in mm, where *e*_*1*_ ≥ *e*_*2*_), and the column length (*L* in mm). These variables are considered to have a direct influence on the axial capacity (*P*_*u*_) of the 777 column observations. As per Naser et al.^23^, additional material properties such as the Young’s modulus of steel (*E*_*s*_) and concrete (*E*_*c*_), and the ultimate strength of steel (*f*_*u*_), have no significant impact on data-driven model training.

**Table 2:** Statistic features of the experimental dataset.

Column cross-section type	Variable	Symbol	Type	Statisticsfoptimi					
				Min	Max	Mean	Std	Skewness	Kurtosis
Circular	Diameter of outer tube	$\:D$ (mm)	Input	76	600	142	53	3.43	21.23
	Thickness of outer tube	$\:t$ (mm)	Input	0.86	16	4.2	1.81	2.2	11.92
	Column length	$\:L$ (mm)	Input	326	4956	1831	1060	0.79	0.32
	Yield strength of outer tube	$\:{f}_{y}$ (MPa)	Input	185.7	517	327.1	60.3	0.45	0.04
	Concrete strength	$\:{f}_{c}^{{\prime\:}}$ (MPa)	Input	15.3	177	48.9	24.9	1.81	4.85
	Section slenderness ratio	$\:{\lambda\:}_{r}$	–	0.02	0.25	0.06	0.03	2.59	9.47
	Length-to-depth ratio	$\:L/D$	–	3	33	13.9	8.1	0.39	-0.69
	Top end eccentricity	$\:{e}_{1}$	Input	5	300	38.8	34.3	3.02	14.36
	Bottom end eccentricity	$\:{e}_{2}$	Input	−50	300	35.2	36.7	2.51	11.45
	Axial load	$\:{P}_{u}$ (kN)	–	96	5288	718	729	3.21	13.47
	Strength index	$\:{p}_{si}$ (MPa)	Output	0.12	1.34	0.51	0.23	0.6	−0.25
Rectangular	Height of outer tube	H (mm)	Input	76	323	155	47	1.29	2.46
	Width of outer tube	B (mm)	Input	76	323	152	45	1.32	2.61
	Thickness of outer tube	$\:t$ (mm)	Input	1.25	12.5	4.62	1.7	1.41	3.18
	Column length	$\:L$ (mm)	Input	330	4500	1778	969	0.5	−0.58
	Yield strength of outer tube	$\:{f}_{y}$ (MPa)	Input	205	1031	379.8	143.4	2.55	7.58
	Concrete strength	$\:{f}_{c}^{{\prime\:}}$ (MPa)	Input	16.9	176	59.4	29.2	0.68	0.44
	Section slenderness ratio	$\:{\lambda\:}_{r}$	–	0.57	6.78	1.54	0.75	2.58	13.84
	Length-to-depth ratio	$\:L/D$	–	2	50	13.1	9.3	1.03	0.61
	Top end eccentricity	$\:{e}_{1}$	Input	0.9	300	40.6	38.4	3.35	15.9
	Bottom end eccentricity	$\:{e}_{2}$	Input	−25	300	37.8	40.4	2.96	13.46
	Axial load	$\:{P}_{u}$ (kN)	–	184	7136	1298	1029	9.25	125.5
	Normalized load	$\:{p}_{si}$ (MPa)	Output	0.07	3.18	0.59	0.43	1.1	4.7

**Fig. 2:**
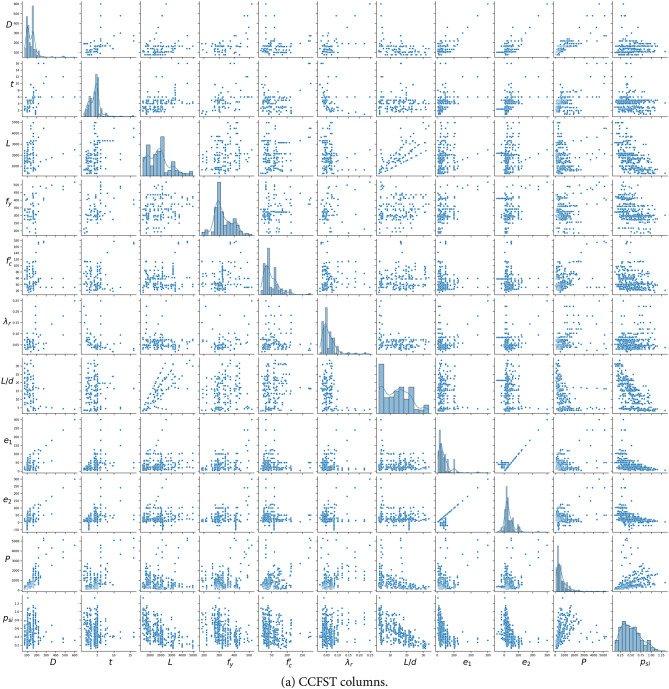

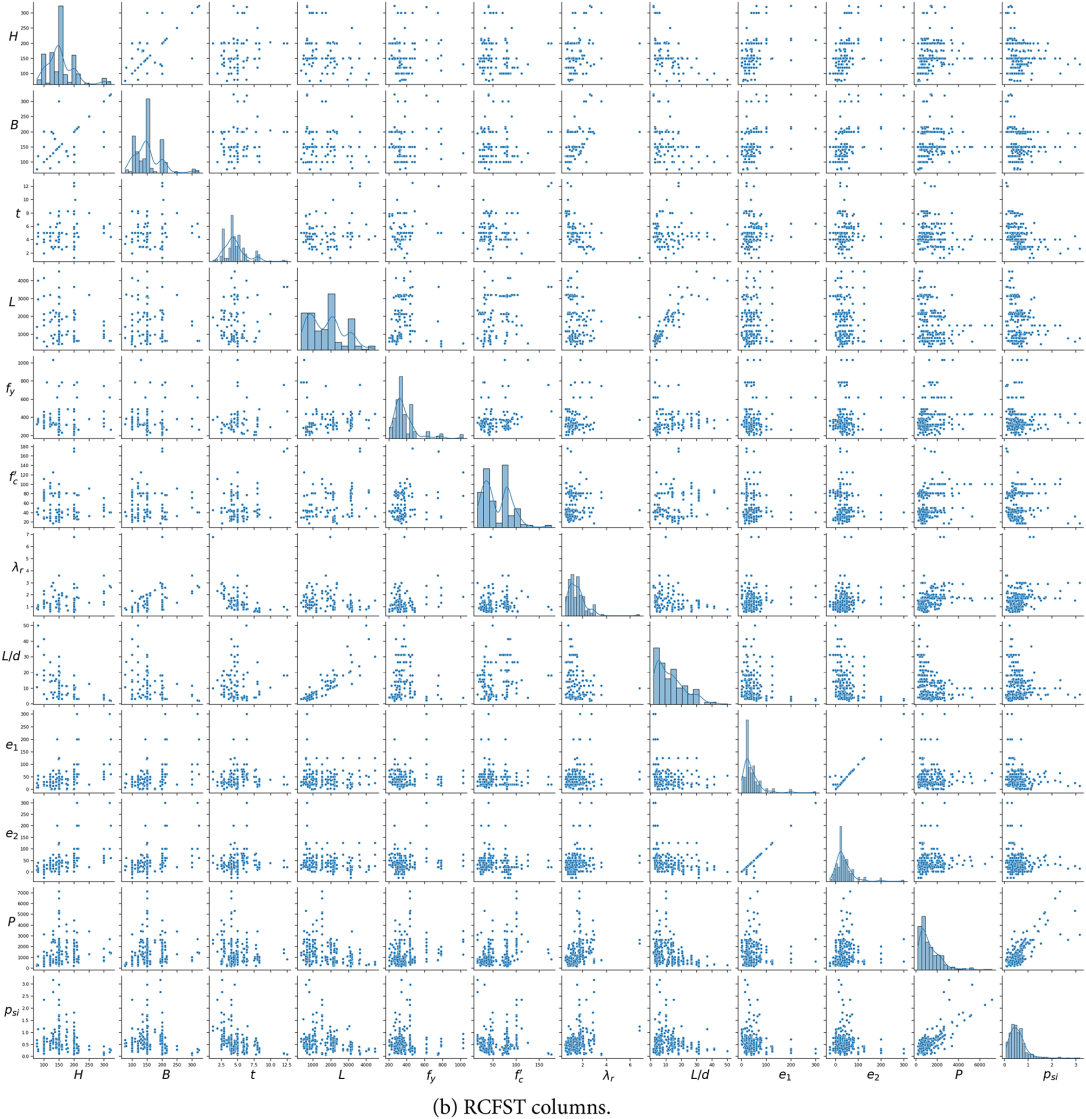
Distribution of the databases and the relationships between different parameters.

The output variable chosen in this study is the dimensionless strength index, denoted as *p*_*si*_. This index is computed by normalizing the eccentric load *P*_*u*_ against the combined strengths of the column’s components, including the steel section and core concrete. It is defined as follows:

1 $${p_{si}}=\frac{{{P_u}}}{{{N_{pl}}}},~~{N_{pl}}={A_s}{f_y}+{A_c}f_{c}^{\prime }$$

where *A*_*s*_ and *A*_*c*_ the areas of the steel and concrete, respectively. Table 2; Fig. 2 provide statistical summaries of the output variable and the 12 input features derived from the databases of CCFST and RCFST columns. The strength index, *p*_*si*_, captures the effects of global column slenderness and load eccentricity. Slender columns, characterized by larger length-to-depth ratios and greater load eccentricity, tend to have lower *p*_*si*_ values. It has been demonstrated that using the strength index (*p*_*si*_) instead of column capacity (*P*_*u*_) as the primary output significantly improves machine learning prediction performance^19,20^.

The most critical parameters influencing column stability are the global slenderness, determined by the length-to-depth ratio, and the local slenderness coefficient, *λ*_*r*_, defined in Eq. (2) for CCFST and RCFST columns^27^:

2 $$\:{\lambda\:}_{r}\:=\frac{D}{t}\left(\frac{{f}_{y}}{{E}_{s}}\right)\:\:\left(circular\right),\:\:{\lambda\:}_{r}=\frac{H}{t}\sqrt{\frac{{f}_{y}}{{E}_{s}}}\:\:\left(\text{r}\text{e}\text{c}\text{t}\text{a}\text{n}\text{g}\text{u}\text{l}\text{a}\text{r}\:\right)$$

As illustrated in Table 2, the database spans a wide range of steel section slenderness, covering compact (λ ≤ 0.15 for CCFST, λ ≤ 2.26 for RCFST), noncompact (0.15 ≤ λ ≤ 0.19 for CCFST, 2.26 ≤ λ ≤ 3.0 for RCFST), and slender (λ > 0.19 for CCFST, λ > 3.0 for RCFST) columns, as suggested by AISC360-22^27^. Furthermore, the database includes a broad spectrum of concrete and steel strengths, ranging from traditional materials (*f*_*c*_*’* < 70 MPa and *f*_*y*_ < 460 MPa^27^) to high-strength classes (with *f*_*c*_*’* up to 177 MPa and *f*_*y*_ up to 1031 MPa).

## Research methodology

### ML algorithms

This study employs six different machine learning (ML) models to predict the eccentric strength of CFST columns: categorical boosting (CatBoost)^28^, extreme gradient boosting (XGBoost)^29^, light gradient-boosting machine (LightGBM)^30^, random forests (RF)^31^, Gaussian Process Regression (GPR)^9^, and support vector regression (SVR)^32^. The performance of these models is evaluated and compared. Typically, ensemble learning methods offer greater accuracy and stability compared to individual models^28^.

CatBoost, LightGBM, and XGBoost are ensemble techniques based on boosting, where multiple weak learners are combined iteratively to create a stronger predictor^33^. CatBoost is particularly effective with categorical data, as it eliminates the need for preprocessing non-numerical features^28^. It uses unbiased boosting to reduce gradient bias and improve generalization, especially when working with categorical variables. LightGBM^30^ adopts a histogram-based approach for data splitting, making it faster and better suited for large datasets. XGBoost^29^, on the other hand, uses a level-wise depth-first strategy, which may be slower than LightGBM but can yield more robust results for specific tasks. Random Forests, introduced by Breiman^31^, is an ensemble method based on bagging. It trains multiple decision trees on different subsets of data and aggregates their outputs through averaging (for regression) or voting (for classification). Important parameters affecting RF performance include the number of trees, maximum features, and tree depth.

### Eurocode 4 design provisions

Eurocode 4 (EC4) [61] outlines a simplified method for designing composite columns with circular or rectangular cross-sections. For columns subjected to eccentric loading *P*_*u*_ with eccentricity *e*_*1*_, EC4 prescribes that the second-order design moment, *M*_*u*_, must not exceed the column’s resisting moment. The second-order design moment is an amplification of the first-order design moment $\:{P}_{u}{e}_{1}$ to account for the additional effects caused by lateral deflection (second-order effects). This amplification is expressed as:

3 $$\:{M}_{u}={P}_{u}{e}_{1}\left[\frac{1.1\beta\:}{1-\frac{{P}_{u}}{{P}_{cr}}}\right]\le\:\chi\:{M}_{n,{p}_{u}},$$

in which:

4 $$\:\beta\:=0.6+0.4\frac{{e}_{2}}{{e}_{1}},\:\:{P}_{cr}=\frac{{\pi\:}^{2}E{I}_{eff}}{{\left(KL\right)}^{2}},\:\:E{I}_{eff}=0.9\left({E}_{c}{I}_{s}+0.6{E}_{c}{I}_{c}\right)$$

In these equations, $\:{E}_{c}{I}_{s}$ and $\:{E}_{c}{I}_{c}$ represent the gross flexural sectional stiffness of the steel and concrete components, respectively. $\:{P}_{cr}$ is the buckling load of the column. $\:{M}_{n,{p}_{u}}$ is the section moment strength corresponding to the axial load $\:{P}_{u}$. EC4 recommends a value of *χ* = 0.9 for steel grades between S235 and S355, while *χ* = 0.8 is used for higher grades like S420 and S460.

**Fig. 3:**
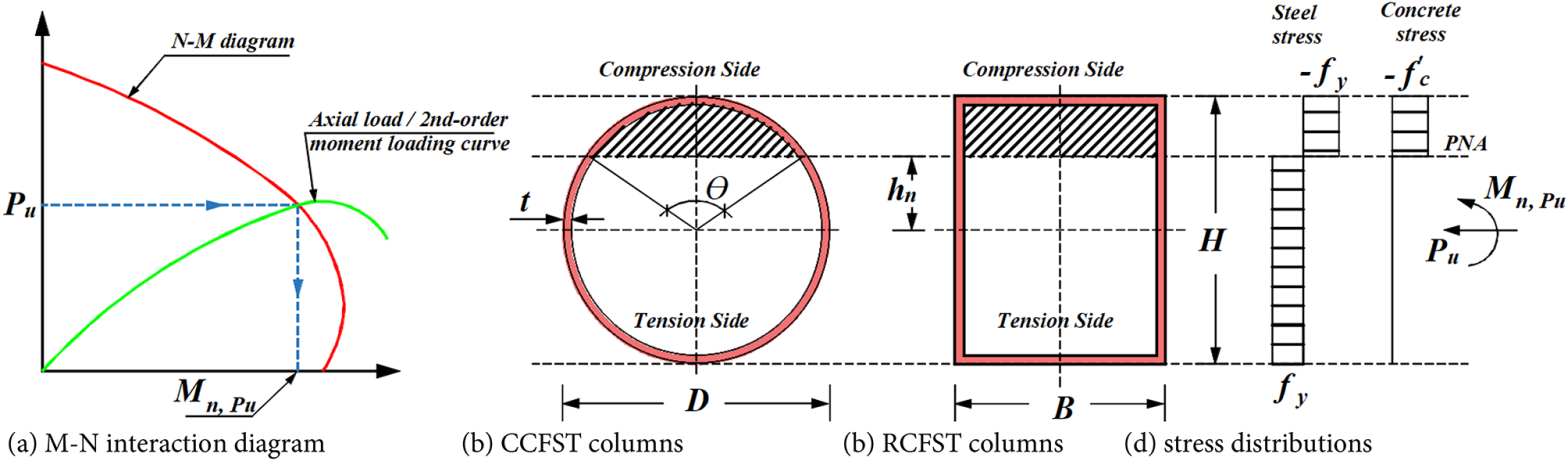
Computation of M-N interaction diagram for CCFST and RCFST columns using plastic stress distributions.

To analyze the resistance of a composite column subjected to a combination of axial load and bending moment $\:\left({P}_{u},{M}_{n,{p}_{u}}\right)$, the interaction between load and moment (N-M interaction diagram) must be determined (Fig. 3). EC4 simplifies the construction of this curve by assuming a rectangular stress block and neglecting the tensile strength of the concrete. Figure 3 illustrates the plastic stress distribution for concrete-filled sections.

For circular concrete-filled steel tube (CCFST) columns, the resisting moment is calculated from load equilibrium as follows:

5 $$\:{M}_{n,{p}_{u}}=\left[{M}_{s}+0.5{M}_{c}\right]{\text{sin}}^{3}\left(\frac{\theta\:}{2}\right),$$

where

6 $$\:{M}_{s}={Z}_{s}{f}_{y},\:\:{M}_{c}={Z}_{c}{f}_{c}^{{\prime\:}}$$

$\:{Z}_{s}$ and $\:{Z}_{c}$ represent the gross plastic section moduli of steel and concrete, respectively. The angle $\:\theta\:$ defines the angle (in radians) subtended by the neutral axis chord to the tube center, as shown in Fig. 3, and is computed by solving:

7 $$\:\theta\:-\text{sin}\theta\:=\rho\:\pi\:\:\:\:\:\:\:\:\:\:\:\:\text{w}\text{i}\text{t}\text{h}\:0<\theta\:<2\pi\:$$

where

8 $$\:\rho\:=\frac{{P}_{u}+{P}_{s}}{0.5{P}_{c}+{P}_{s}},\:\:{P}_{c}={A}_{c}{f}_{c}^{{\prime\:}},\:\:{P}_{s}={A}_{s}{f}_{y}$$

Here, $\:{P}_{s}={f}_{y}{A}_{s}$, $\:{P}_{c}={f}_{c}^{{\prime\:}}{A}_{c}$, and $\:{A}_{s},{A}_{c}$ are the cross-sectional areas of steel and concrete, respectively. The closed-form expressions presented in Eqs. (5), (6), (7), (8) are derived from integrating the stress distribution and applying force equilibrium. Its derivation is detailed in the supplementary data.

For rectangular concrete-filled steel tube (RCFST) columns, the resisting moment is defined by a similar approach, as shown below:

9 $$\:{M}_{n,{p}_{u}}=\left[{M}_{s}-{b}_{s}{h}_{n}^{2}\right]+0.5\left[{M}_{c}-{b}_{c}{h}_{n}^{2}\right]$$

where *h*_*n*_ is the plastic neutral axis location (see Fig. 3), calculated as:

10 $$\:{h}_{n}=\frac{{P}_{u}-0.5{P}_{c}}{{b}_{c}+2{b}_{s}},$$

where $\:{b}_{c}={f}_{c}^{{\prime\:}}\left(b-2t\right)$, $\:{b}_{s}={f}_{y}\left(2t\right).$ Using these expressions, the N-M interaction curve and the axial load-to-second-order moment curve can be constructed. As shown in Fig. 3, the intersection of the load and resistance curves represents the predicted resistance of the eccentrically loaded CFST column.

### Symbolic regression

Symbolic regression (SR)^34,35^ is a genetic programming technique^11^ designed to discover simple, interpretable equations that best represent a given problem by exploring a predefined space of mathematical expressions. It is considered a multi-objective optimization problem, balancing the trade-off between predictive accuracy and model complexity. Using natural selection and evolution principles, SR iteratively refines candidate mathematical models, searching for the most satisfactory solutions. In this study, the Python library PySR^36^ is employed to identify concise and interpretable expressions for predicting the compressive strength of CFST columns under eccentric loading.

**Fig. 4:**
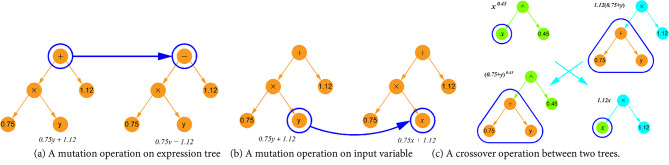
Mutation and crossover operations in SR model.

**Fig. 5:**
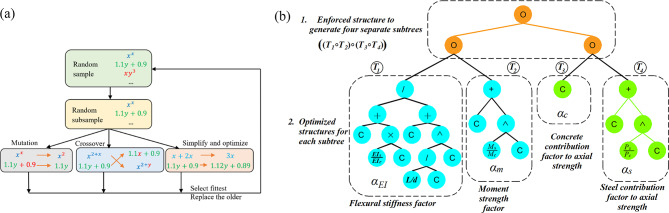
(**a**) Symbolic regression flow chart. (**b**) The optimal tree-based individuals for CCFST columns, where operator O, in the forced structure part, can be any arbitrary binary operator. Variable C in the optimized structure part is constant.

The SR algorithm begins by generating an initial population of mathematical expressions composed of operational symbols (e.g., +, -, *,/, ^, etc.) and terminals such as input variables and constants. Each individual expression is structured as a tree. Selection is then performed probabilistically, favoring expressions with superior performance. To avoid excessive complexity, a complexity limit of 30 for the total number of nodes is set, meaning the total number of operators, constants, and variables in any expression cannot exceed this value. Furthermore, overly complex terms, like high-order exponentials (e.g. (•)^(•^•)), are excluded. Selected expressions then undergo mutation or crossover (as shown in Fig. 4) to generate new populations for the next generation. Figure 5a presents the core steps of the SR approach. This evolutionary process is guided by a fitness function, which balances prediction accuracy with simplicity. The fitness function is defined as:

11 $$\:l\left(E\right)={l}_{pred}\left(E\right).\text{exp}\left(\text{f}\text{r}\text{e}\text{c}\text{e}\text{n}\text{c}\text{y}\left[C\left(E\right)\right]\right)$$

where *l*_*pred*_(*E*) and *C*(*E*) define, respectively, the prediction error and the expression complexity *E*, quantified by the total number of nodes in the expression. The frecency [*C*(*E*)] for how often an expression of complexity *C*(*E*) occurs. This measure is crucial for avoiding overcomplicated and redundant expressions, ensuring a balance between minimizing error and maintaining simplicity. Details of the SR parameters used for generating expressions in this study are summarized in Table 3.

**Table 3:** The parameters of the SR model used in generating expressions.

Parameters	Value	Parameters	Value
Number of generations	200	Allowed binary operators	−, +, *, ^, /
Total number of populations	40	Loss function	Algorithm 1
Population size	50	Constraints	{‘^’:(–1,10)}^(a)^
Maximum length of expressions (total number of nodes)	80	Nested constraints	{“^”: {“^”: 0, “/“: 0}}^(b)^
Parsimony (factor controls the expression complexity)	0.02	model_selection	Accuracy

(a)The ‘^’:(–1,10) constraint means that the left argument of the power function can exhibit any level of complexity, whereas the right argument is restricted to a maximum complexity of 10 nodes.

(b)Nested constraints govern how operators can be combined or nested. The constraint ‘^’:{‘^’:0,’/’:0} specifies that ‘^’ or ‘/’ operator cannot be used inside another ‘^’ operator.

**Algorithm 1:**
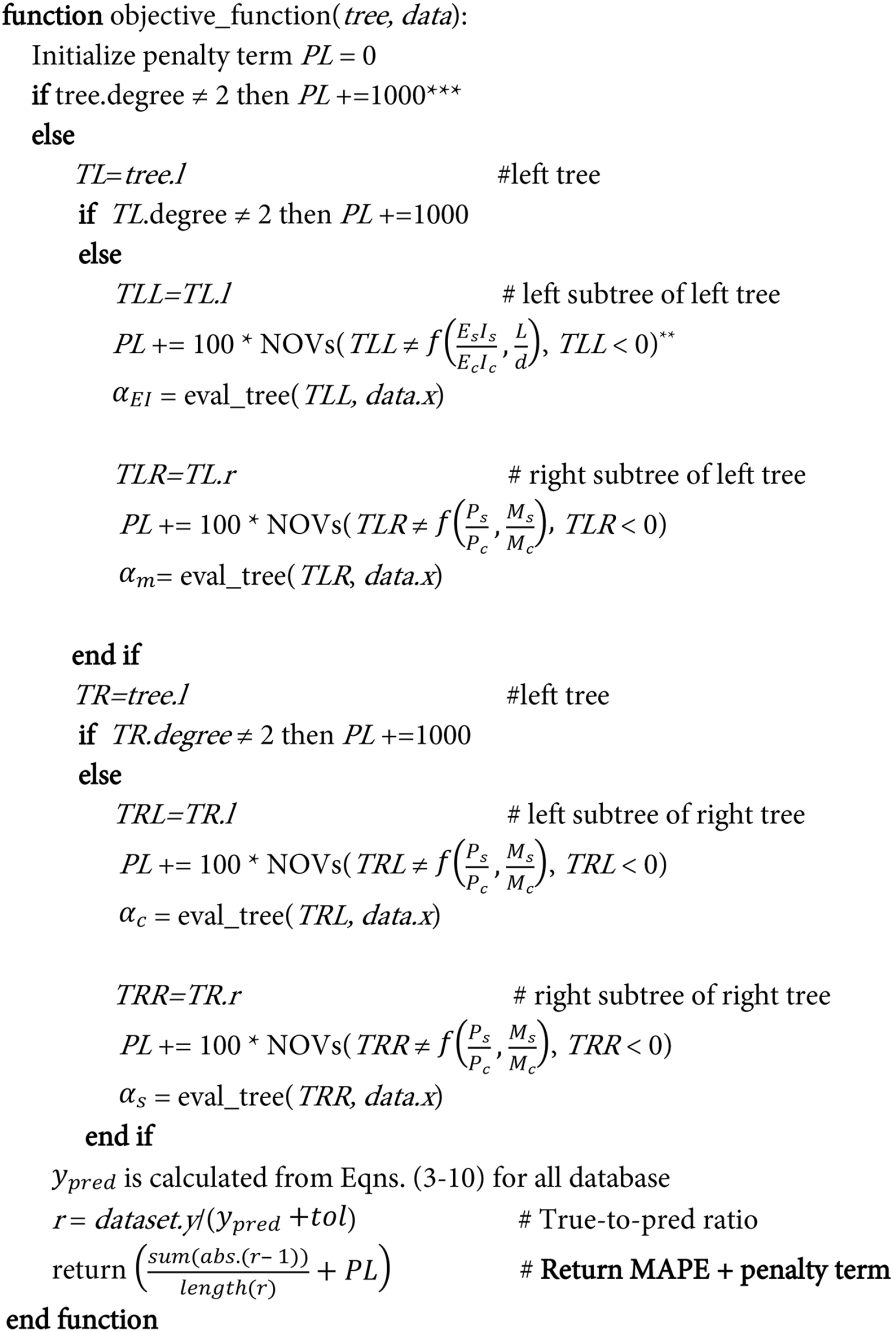
Objective function. **The expression NOV(TLL ≠ && TLL < 0) quantifies the number of violations where the TLL subtree is not a function of the features and or where it includes other features, or when its value is negative. ***The penalty values used in the loss function is recommended by PySR^36^ (see the discussion https://github.com/MilesCranmer/PySR/discussions/557).

During the training process, the complexity of the expressions was carefully managed through several mechanisms. First, the population initialization involved generating an initial set of expressions with varying complexities, but all were capped at the defined limit of 30 nodes. As the evolutionary process progressed, mutation and crossover operations were applied to create new expressions while ensuring that the complexity constraint was not violated; any mutations resulting in overly complex expressions were discarded. The selection process further controlled complexity by probabilistically favoring expressions with better fitness scores, which inherently balanced accuracy and simplicity. This ensured that simpler yet accurate expressions had a higher likelihood of being selected for the next generation.

The search for optimal expressions involves numerous iterations, each producing potential solutions. During this process, factors such as model complexity, prediction accuracy, and ease of interpretation are carefully evaluated and refined. By varying key parameters like population size, number of generations, and mutation rate, different equations are generated and assessed, ensuring that the final model offers a balance between simplicity and predictive power. This iterative refinement guarantees that the selected model is both interpretable and accurate.

Machine learning models present a robust and innovative approach for predicting the eccentric capacity of CFST columns. While some existing machine learning models and simplified design equations have been introduced for CFST column predictions^14,15,24,37^, further research is essential for the following reasons: As noted in the literature in Table 1, the predicted formulas for designing CFST columns using genetic algorithms (GA) and Genetic Programming (GEP) demonstrate efficiency and compatibility with experimental results. However, many provided formulas are complicated, unit-dependent, and lack adequate explanations. This paper introduces a novel model to derive simple, interpretable, and unit-independent expressions for predicting the compression strength of CFST columns under eccentric loading.

## Code-based symbolic regression development

The primary aim of this section is to utilize symbolic regression (SR) to refine and calibrate the code standards for predicting the eccentric capacity of CCFST and RCFST columns. Unlike purely data-driven approaches, where machine learning models directly derive predictions from data, the SR-based calibration method presented here integrates the provisions of code standards with machine learning techniques to enhance prediction accuracy. This hybrid approach combines the physical insights embedded in code standards with the optimization capabilities of symbolic regression, yielding precise and meaningful prediction outcomes.

The design standard codes, including AISC360^27^, AIJ^38^ and AS/NZS 2327^39^ are similar in conception to EC4^26^. However, they differ in their definitions of various key variables, such as effective flexural stiffness, $\:{\stackrel{-}{EI}}_{eff}$, the contributions of steel and concrete axial strengths $\:{\stackrel{-}{P}}_{s}$, $\:{\stackrel{-}{P}}_{c}$ and the concrete bending strength $\:{\stackrel{-}{M}}_{c}$, as defined previously in EC4, Eqs. (4), (8), (6), respectively. Symbolic regression is employed to accurately compute these four contributions through separate subtrees, expressed as:

12 $$\:{\stackrel{-}{EI}}_{eff}={\alpha\:}_{EI}{E}_{c}{I}_{c},\:\:{\stackrel{-}{P}}_{s}={\alpha\:}_{s}{P}_{s},\:\:{\stackrel{-}{P}}_{c}={\alpha\:}_{c}{P}_{c},\:\:{\stackrel{-}{M}}_{c}={\alpha\:}_{m}{M}_{c}$$

Here, $\:{\alpha\:}_{EI}$, $\:{\alpha\:}_{s}$, $\:{\alpha\:}_{c}$, and $\:{\alpha\:}_{m}$ define the contribution factors for the variables $\:{\stackrel{-}{EI}}_{eff}$, $\:{\stackrel{-}{P}}_{s}$, $\:{\stackrel{-}{P}}_{c}$, and $\:{\stackrel{-}{M}}_{c}$, respectively, defined as follows:

13 $$\:{\alpha\:}_{EI}={\alpha\:}_{EI}\left(\frac{{E}_{s}{I}_{s}}{{E}_{c}{I}_{c}},\frac{L}{d}\right),\:\:{\alpha\:}_{s}={\alpha\:}_{s}\left(\frac{{P}_{s}}{{P}_{c}},\frac{{M}_{s}}{{M}_{c}}\right),\:\:{\alpha\:}_{c}={\alpha\:}_{c}\left(\frac{{P}_{s}}{{P}_{c}},\frac{{M}_{s}}{{M}_{c}}\right),\:\:{\alpha\:}_{m}={\alpha\:}_{m}\left(\frac{{P}_{s}}{{P}_{c}},\frac{{M}_{s}}{{M}_{c}}\right)$$

These factors are functions of dimensionless variables. For instance, the effective flexural stiffness factor $\:{\alpha\:}_{EI}$ is chosen as a function of $\:\left(\frac{{E}_{s}{I}_{s}}{{E}_{c}{I}_{c}}\right)$ and $\:\frac{L}{d}$ as these variables control the buckling load of the column, $\:{P}_{cr}$. The depth *d* refers to the column depth in the loading direction (with *d* = *h* for RCFST columns and *d = D* for CCFST columns). The remaining factors are chosen to be a function of $\:\frac{{P}_{s}}{{P}_{c}}$ and$\:\:\frac{{M}_{s}}{{M}_{c}}$ as these ratios reflect the confinement effect of steel tube on the core concrete and local buckling slenderness effect. Increasing the relative strength of the steel tube enhances the confinement effect on the compressive strength of the core concrete due to the impact of the triaxial compressive stresses while simultaneously reducing local buckling slenderness^1,2,40^.

In this study, symbolic regression is utilized to refine the coefficient expressions for various components of the code standards: the effective stiffness (*EI*_*eff*_), concrete and steel contribution to axial strength (*P*_*c*_, *P*_*s*_), and concrete contribution to bending strength *M*_*c*_. This involves developing a custom objective loss function tailored specifically for symbolic regression, which predefines the structure of the computational procedures of column strength along with the four expressions to be optimized. In this process, the accepted symbolic trees are chosen to be limited to the enforced structure of $\:\left(\left({T}_{1}\circ\:{T}_{2}\right)\circ\:\left({T}_{3}\circ\:{T}_{4}\right)\right)$, where subtrees *T*_*1*_, *T*_*2*_, *T*_*3*_, and *T*_*4*_ define the four functions $\:{\alpha\:}_{EI}$, $\:{\alpha\:}_{m}$, $\:{\alpha\:}_{c}$, and $\:{\alpha\:}_{s}$, respectively. The operator ∘ can represent any arbitrary binary operator. This structure, referred to as the enforced tree structure generated by the developed objective function, is detailed in Fig. 5b. The details of the objective function are outlined in Algorithm 1. The objective function imposes constraints on the structure of the symbolic expressions and penalizes undesirable characteristics.

As described in Algorithm 1, the degree of the tree head is checked; if it does not equal two (indicating that it should combine two subtrees), a substantial penalty of 1000 is applied. Both the left subtree (TL = $\:\left({T}_{1}\circ\:{T}_{2}\right)$) and the right subtree (TR = $\:\left({T}_{3}\circ\:{T}_{4}\right)$) are also examined for a degree of two, with a smaller penalty of 100 imposed if they do not meet this criterion. The left child of the left subtree (TLL or *T*_*1*_) represents $\:{\alpha\:}_{EI}$ (effective flexural stiffness factor) and must be a function of $\:\left(\frac{{E}_{s}{I}_{s}}{{E}_{c}{I}_{c}}\right)$ and $\:\frac{L}{d}$. If $\:{\alpha\:}_{EI}$ includes invalid features or negative values, penalties are applied in proportion to the number of violations. Similarly, the right child of the left subtree (TLR or *T*_*2*_) is processed as $\:{\alpha\:}_{m}$ (concrete bending strength factor), which should only depend on $\:\frac{{P}_{s}}{{P}_{c}}$ and$\:\:\frac{{M}_{s}}{{M}_{c}}$. Similarly, the right subtree (TR) must also branch into two children (TRL, TRR) or (*T*_*3*_, *T*_*4*_), representing the contribution factors for steel and concrete axial strengths, respectively. It should be noted that the penalty term increases progressively by how far it deviates from the constraints, effectively guiding the genetic algorithm toward the desired expression. Finally, the loss function sums up the penalty term and the Mean Absolute Percentage Error (MAPE) of the predictions.

The optimal tree-based individuals (Fig. 5b) fitting the training experimental database for CCFST columns are expressed as:

14 $$\:{\alpha\:}_{EI}=\frac{1.0+0.311\left(\frac{{E}_{s}{I}_{s}}{{E}_{c}{I}_{c}}\right)}{0.54+{\left(\frac{L}{10.4D}\right)}^{-0.736}},\:\:{\alpha\:}_{m}=0.7+{\left(\frac{{M}_{s}}{5.8{M}_{c}}\right)}^{1.31},\:\:{\alpha\:}_{c}=1.0,\:\:{\alpha\:}_{s}=0.8+{\left(\frac{{P}_{s}}{13.4{P}_{c}}\right)}^{0.19}\ge\:1.25,$$

and for RCFST columns as:

15 $$\alpha _{{EI}} = 0.87\left[ {\frac{{E_{s} I_{s} }}{{E_{c} I_{c} }} + \left( {0.49 + \left( {\frac{L}{{3.4h}}} \right)^{{0.8}} } \right)} \right],~~\alpha _{m} = 0.67 + 0.36\left( {\frac{{M_{s} }}{{M_{c} }}} \right)^{{ - 0.15}} ,~~\alpha _{c} = 0.98 - 0.13\left( {\frac{{P_{s} }}{{P_{c} }}} \right)^{{3.0}} > 0.4~,\alpha _{s} = 1.34.$$

Table 4 summarizes the proposed design and EC4 standards.

The developed expressions for the code-based symbolic regression (C-SR) model are not only straightforward and robust but also carry physical significance, in contrast to the GEP and GA models from previous studies (see Table 1). Furthermore, the enforced structure tree for symbolic regression narrows the search space and ensures that the resulting expressions are both explainable and consistent with the established code provisions.

**Table 4:** Summary of EC4 provisions with the proposed design.

	Formulas
EC4^26^	$\:{M}_{u}={P}_{u}{e}_{1}\left[\frac{1.1\beta\:}{1-\frac{{P}_{u}}{{P}_{cr}}}\right]\le\:\chi\:{M}_{n,{p}_{u}}$ $\:\:\beta\:=0.6+0.4\frac{{e}_{2}}{{e}_{1}},\:\:{P}_{cr}=\frac{{\pi\:}^{2}E{I}_{eff}}{{\left(KL\right)}^{2}},\:\:E{I}_{eff}=0.9\left({E}_{c}{I}_{s}+0.6{E}_{c}{I}_{c}\right),\:\chi\:=0.9\:for\:S235,S355,\chi\:=0.8\:for\:S\text{420,460}$
	CCFST columns: $\:{M}_{n,{p}_{u}}=\left[{M}_{s}+0.5{M}_{c}\right]{\text{sin}}^{3}\left(\frac{\theta\:}{2}\right)$ $\:\text{S}\text{o}\text{l}\text{v}\text{e}\:\theta\:-\text{sin}\theta\:=\rho\:\pi\:\text{f}\text{o}\text{r}\:0<\theta\:<2\pi\:$ $\:\rho\:=\frac{{P}_{u}+{P}_{s}}{0.5{P}_{c}+{P}_{s}},\:\:{P}_{c}={A}_{c}{f}_{c}^{{\prime\:}},\:\:{P}_{s}={A}_{s}{f}_{y},\:\:{M}_{s}={Z}_{s}{f}_{y},\:\:{M}_{c}={Z}_{c}{f}_{c}^{{\prime\:}}$
	RCFST columns: $\:{M}_{n,{p}_{u}}=\left[{M}_{s}-{b}_{s}{h}_{n}^{2}\right]+0.5\left[{M}_{c}-{b}_{c}{h}_{n}^{2}\right],\:\:{h}_{n}=\frac{{P}_{u}-0.5{P}_{c}}{{b}_{c}+2{b}_{s}},{b}_{c}={f}_{c}^{{\prime\:}}\left(b-2t\right),{b}_{s}={f}_{y}\left(2t\right)$
Proposed design	$\:{M}_{u}={P}_{u}{e}_{1}\left[\frac{\beta\:}{1-\frac{{P}_{u}}{{P}_{cr}}}\right]\le\:\chi\:{M}_{n,{p}_{u}}$
	CCFST columns: $\:E{I}_{eff}=\frac{{E}_{c}{I}_{c}+0.311{E}_{s}{I}_{s}}{0.54+{\left(\frac{L}{10.4D}\right)}^{-0.736}}$ $\:{M}_{n,{p}_{u}}=\left[{M}_{s}+0.5{\alpha\:}_{m}{M}_{c}\right]{\text{sin}}^{3}\left(\frac{\theta\:}{2}\right),\:\:{\alpha\:}_{m}=0.7+{\left(\frac{{M}_{s}}{5.8{M}_{c}}\right)}^{1.31},\:\:\chi\:=1.0$ $\:\text{S}\text{o}\text{l}\text{v}\text{e}\:\theta\:-\text{sin}\theta\:=\rho\:\pi\:\:\:\text{f}\text{o}\text{r}\:0<\theta\:<2\pi\:$ $\:\rho\:=\frac{{P}_{u}+{P}_{s}{\alpha\:}_{s}}{0.5{P}_{c}+{P}_{s}{\alpha\:}_{s}},\:\:{\alpha\:}_{s}=0.8+{\left(\frac{{P}_{s}}{13.4{P}_{c}}\right)}^{0.19}\ge\:1.25$
	RCFST columns: $EI_{{eff}} = 0.87\left[ {E_{s} I_{s} + E_{c} I_{c} \left( {0.49 + \left( {\frac{L}{{3.4h}}} \right)^{{0.8}} } \right)} \right]$ $\:{M}_{n,{p}_{u}}=\left[{M}_{s}-{b}_{s}{h}_{n}^{2}\right]+0.5{\alpha\:}_{m}\left[{M}_{c}-{b}_{c}{h}_{n}^{2}\right],\:\:{\alpha\:}_{m}=0.67+0.36{\left(\frac{{M}_{s}}{{M}_{c}}\right)}^{-0.15},\:\:\chi\:=0.87$ $\:{h}_{n}=\frac{{P}_{u}-{\alpha\:}_{c}\left(0.5{P}_{c}\right)}{{\alpha\:}_{c}{b}_{c}+2{\alpha\:}_{s}{b}_{s}},{b}_{c}={f}_{c}^{{\prime\:}}\left(b-2t\right),{b}_{s}={f}_{y}\left(2t\right)$ $\:{\alpha\:}_{s}=1.34,\:\:{\alpha\:}_{c}=0.98-0.13{\left(\frac{{P}_{s}}{{P}_{c}}\right)}^{3.0}>0.4$

**Fig. 6:**
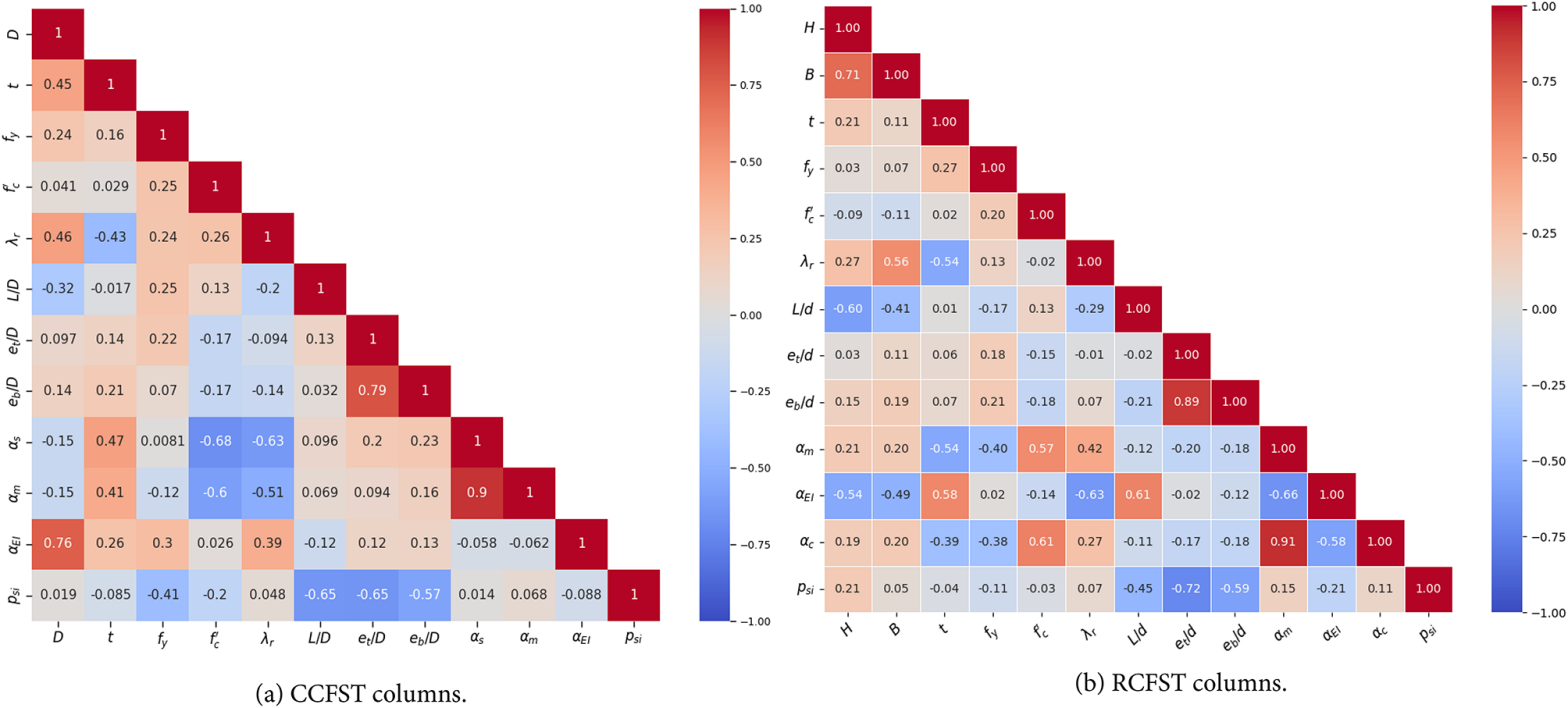
Correlation matrix for variables of CFST columns.

The correlation matrix^41^ in Fig. 6 provides a clear overview of the relationships between the studied parameters, ranging from − 1 to + 1. Strong positive or negative correlations indicate significant linear relationships, while near-zero correlations suggest minimal association. For CCFST columns, regarding the elastic stiffness contribution factor $\:{\alpha\:}_{EI}$, it exhibits a strong positive correlation with the diameter (D), with a correlation coefficient (r) of 0.76. This indicates that larger sections significantly increase the elastic stiffness due to their geometry as derived in Eq. (14). Similarly, there is a moderate positive correlation with thickness (t) (*r* = 0.26), highlighting that thicker sections enhance stiffness parameter. The moment contribution factor ($\:{\alpha\:}_{m}$) shows a strong positive correlation with the steel strength contribution factor ($\:{\alpha\:}_{s}$) (*r* = 0.9), reflecting their dependence on the ratio of steel to concrete strength as explained in Eq. (14). Both parameters exhibit a moderate positive correlation with thickness (t) (*r* = 0.41 for $\:{\alpha\:}_{m}$ and *r* = 0.47 for $\:{\alpha\:}_{s}$), indicating that increasing section thickness improves both bending and axial capacities. On the other hand, the slenderness ratio ($\:{\lambda\:}_{r}$) has a negative correlation with $\:{\alpha\:}_{m}$ (*r* = − 0.51) and $\:{\alpha\:}_{s}$ (*r* = − 0.63), demonstrating that slender sections are less effective in resisting bending and axial forces. The strength index, *p*_*si*_ is notably affected by relative parameters such as the eccentricity ratio (et/D), length-to-diameter ratio (L/D) and slenderness ($\:{\lambda\:}_{r}$), with a moderate negative correlation (*r* = − 0.65, *r* = − 0.65, and *r* = − 0.57, respectively), indicating that higher eccentricities or slenderness ratios reduce axial load performance. For RCFST columns, a similar conclusion can be drawn, with the exception of the slenderness ratio ($\:{\lambda\:}_{r}$), which has a negligible effect on the strength index. This is attributed to the limited confinement provided by the rectangular sections.

## Data preprocessing and hyperparameter optimization using bayesian techniques

In this study, data normalization is performed using the min-max scaling technique to address challenges related to multidimensionality. After normalization, the dataset is randomly split into two subsets, with 80% allocated for training and the remaining 20% reserved for testing.

The performance of machine learning (ML) models is heavily influenced by the selection of hyperparameters, which must be configured prior to training. Proper tuning of these hyperparameters is crucial for achieving optimal predictive performance. The process involves exploring various combinations of hyperparameters to find the best configuration based on validation data. Traditional methods, such as grid search (GS) and random search (RS), can be exhaustive and time-consuming, especially for models with many hyperparameters and large search spaces. In contrast, Bayesian Optimization (BO) utilizes surrogate models, like Gaussian processes or tree-structured Parzen estimators (TPE)^42^, to intelligently guide the search for the next hyperparameter set based on the performance of previous configurations. This reduces redundant evaluations and enables BO to find the optimal hyperparameter settings with fewer iterations compared to GS and RS^43^. For this study, the TPE model was chosen to optimize the ML models’hyperparameters due to its robustness and efficiency compared to other surrogate models^43^.

## Performance and results of ML models

**Fig. 7:**
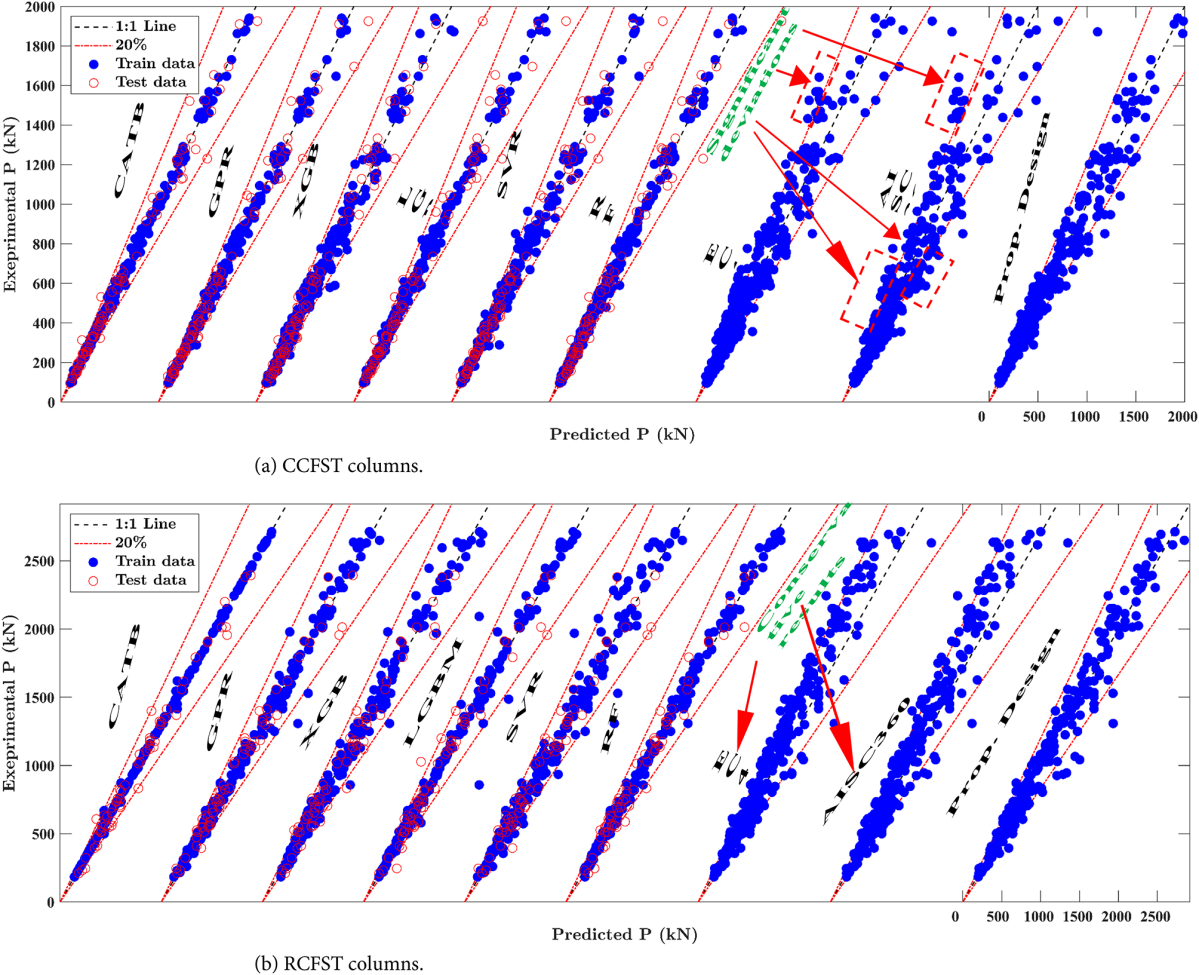
Comparison between proposed design and previous models.

**Fig. 8:**
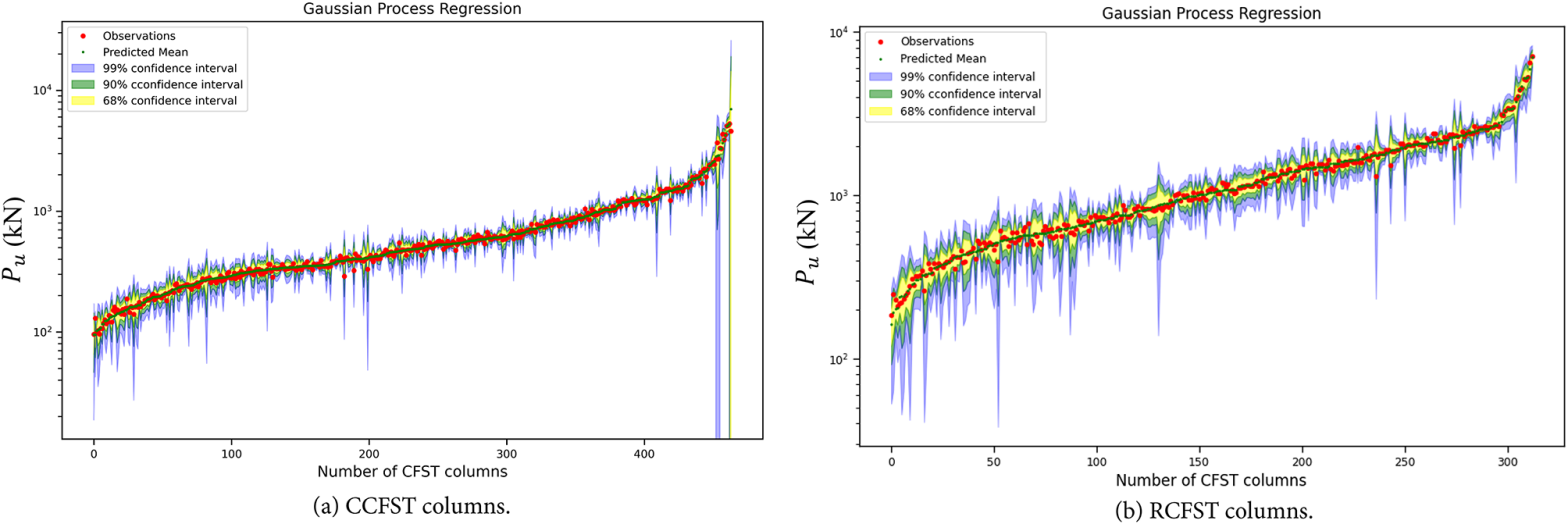
Gaussian process regression on a semilog scale on the y-axis for CFST columns.

This section provides a detailed performance comparison of the machine learning (ML) models introduced in the study. The details of the developed ML models are provided in the supplementary data, including hyperparameter tuning processes and results. The scatter plots in Fig. 7 depict the relationship between experimental and predicted strength for different ML models applied to the training and testing datasets for CCFST and RCFST columns. In most cases, data points are tightly clustered around the diagonal line, demonstrating a strong correlation between model predictions and experimental results. This strong alignment highlights the accuracy and reliability of the developed ML models. In addition to the high accuracy of the GPR model high accuracy, as plotted in Fig. 7, it can also provide confidence intervals for its predictions, as demonstrated in Fig. 8. This ability to quantify uncertainty increases its usefulness in guiding practical design decisions by providing the confidence levels of the predictions made.

Tables 5 and 6 outline several key evaluation metrics used to assess the performance of these ML models: (1) mean (*µ*), which defines the ratio between actual and predicted values, offering insight into overall model accuracy; (2) coefficient of variance (CoV), which reflects the variability of predictions relative to the mean, (3) coefficient of determination (*R*^*2*^), which indicates the proportion of variance in the dependent variable that is explained by the model, (4) root mean squared error (RMSE), which captures the average error in predictions, with a focus on more significant errors, (5) the mean absolute percentage error (MAPE), which evaluates the percentage error between actual and predicted values, and (6) a20-index^16^, which represents the percentage of predictions where the ratio of actual to predicted values lies between 0.80 and 1.20. The formulas for each of these metrics are provided as follows:

16 $$\:\mu\:=\frac{1}{n}\sum\:_{i=1}^{n}\frac{{y}_{i}}{{\widehat{y}}_{i}},\:\:{R}^{2}=1-\frac{\sum\:_{i=1}^{n}{\left({\widehat{y}}_{i}-{y}_{i}\right)}^{2}}{\sum\:_{i=1}^{n}{\left({y}_{i}-\stackrel{-}{y}\right)}^{2}}\:\:RMSE=\sqrt{\frac{1}{n}\sum\:_{i=1}^{n}{\left({\widehat{y}}_{i}-{y}_{i}\right)}^{2}},\:\:MAPE=\frac{1}{n}\sum\:_{i=1}^{n}\left|\frac{{y}_{i}}{{\widehat{y}}_{i}}-1\right|\times\:100\%$$

where $\:{y}_{i}$ and $\:{\widehat{y}}_{i}$ are the actual experimental and predicted strength values of the *i-*th sample, respectively, $\:\stackrel{-}{y}$ is the mean value of experimental output results, and *n* is the number of specimens in the database.

The evaluation metrics presented in Tables 4 and 5 highlight the robust performance of all machine learning (ML) models across both CCFST and RCFST columns. For both column types, the mean *µ*, *R*^*2*^, and a20-index values for CatBoost (CatB), GPR, and LightGBM (LGBM) models are close to 1.0, with small CoV, MAPE%, and RMSE values. This indicates a high degree of accuracy and minimal deviation in predictions compared to the experimental results. As noticed, the CoV for these models is less than 0.083, and MAPE% values are lower than 4.63, indicating minimized scattering in the prediction results compared to the experimental results.

**Table 5:** Comparison of the developed ML models for CCFSTs.

Metrics	Training data			Testing data				All data					
	CatB	GPR	LGBM	CatB		GPR	LGBM	CatB	GPR	LGBM	EC4 ^26^	AISC ^27^	Prop. design
Mean $\:\mu\:$	0.999	0.998	0.999	1.002		0.999	1.001	1	0.999	1	1.041	0.982	1.006
CoV	0.035	0.044	0.05	0.093		0.096	0.104	0.052	0.058	0.065	0.16	0.17	0.117
R^2^	0.999	0.998	0.997	0.97		0.899	0.969	0.99	0.969	0.989	0.954	0.959	0.957
MAPE	2.527	3.173	3.434	6.376		6.738	7.367	3.298	3.887	4.222	13.374	13.884	9.223
RMSE(kN)	22.1	26.4	35.7	154.1		281.9	156.3	71.8	128.4	76.9	156.7	148.1	151.1
a20-index	0.997	0.997	0.995	0.968		0.957	0.957	0.991	0.989	0.987	0.816	0.755	0.92
Time (s)*	0.859	16.72	0.178	0.366		20.69	1.23	–	–	–	–	–	–

*The times in the training data columns refer to the training time. While the times in the testing data columns refer to the inference time for 10^5^ specimens.

**Table 6:** Comparison of the developed ML models for RCFSTs.

Metrics	Training data			Testing data			All data					
	CatB	GPR	LGBM	CatB	GPR	LGBM	CatB	GPR	LGBM	EC4^26^	AISC^27^	Prop. design
Mean $\:\mu\:$	1	1	1.009	0.993	1.014	1.007	0.999	1.003	1.009	1.063	1.051	0.997
CoV	0.024	0.058	0.07	0.097	0.084	0.122	0.048	0.064	0.083	0.134	0.129	0.098
R^2^	1	0.995	0.983	0.975	0.986	0.958	0.995	0.993	0.978	0.956	0.961	0.961
MAPE	0.871	4.101	3.736	6.422	6.708	8.038	1.989	4.625	4.602	11.692	11.004	7.439
RMSE(kN)	18.6	68.2	129.5	157.7	118.7	206.6	72.7	81	148.3	208.8	196.8	196.5
a20-index	1	0.996	0.976	0.952	0.968	0.937	0.99	0.99	0.968	0.815	0.847	0.946
Time (s)*	0.85	3.73	1.14	0.49	6.15	1.42	–	–	–	–	–	–

*The times in the training data columns refer to the training time. While the times in the testing data columns refer to the inference time for 10^5^ specimens.

For CCFST columns, the CatB model exhibits the best performance with MAPE% values of 2.53 for the training set and 6.38 for the testing set. The GPR and LGBM models also perform well, with MAPE% values of 3.17 and 3.43 for training and 6.74 and 7.37 for testing, respectively. All models demonstrate CoV values below 0.065, suggesting minimal scattering in their predictions.

Comparable performance is observed for RCFST columns as well. Although the CatB model for RCFST columns exhibits slightly higher errors for the testing data, achieving a MAPE% of 6.42 compared to the training set error with a MAPE% of 0.87, its overall performance, as measured by remaining evolution metrics, is comparable to other ML models. For instance, it achieves *µ* values of 1.00 and 0.993, *R²* values of 1.00 and 0.975, and a20-index values of 1.00 and 0.952 for training and testing sets, respectively, indicating high accuracy and balance between the two datasets. Such evaluation metrics reveal that the CatB model introduces the best prediction accuracy and predictive balance between the training and testing sets.

When comparing the ML models with the proposed design, the proposed design achieves a mean *µ* of 1.006, a CoV of 0.117 for CCFST columns, and a mean *µ* of 0.997 and a CoV of 0.098 for RCFST columns. Although the accuracy of the proposed design is slightly lower than that of CatB, GPR, and LGBM, its results are comparable to the remaining ML models. Additionally, the proposed design offers better interpretability, making it more practical for real-world applications, in contrast to the black-box nature of many ML models.

In addition, Tables 5 and 6 compare the training and inference times of LGBM, CatB, and GPR models. LGBM model is the fastest for both training and inference for CCFST columns, taking 0.178 s to train and 1.23 s for inference. While CatB model excels for RCFST columns, being the quickest for both training (0.85 s) and inference (0.49 s). GPR model, however, is much slower, requiring 16.72 s for training and 20.69 s for inference. Similar conclusions can be drawn from Table 6. The large time required by GPR model, particularly during training on large datasets, is due to its complex computations, which cause it to scale poorly as the dataset size increases. Thus, while LGBM and CatB models offer more efficient performance, GPR model struggles with larger datasets, making it less suitable for time-sensitive applications.

Tables 5 and 6 also compare the proposed design with existing design codes, including EC4^26^ and AISC360^27^. The proposed design shows significantly better accuracy, with MAPE% values of 9.22 and 7.44 for CCFST and RCFST columns, respectively. In contrast, EC4 and AISC360 present higher errors, with CoV values of 0.16 and 0.17 and MAPE% values of 13.37 and 13.88 for CCFST columns. For RCFST columns, EC4 and AISC360 exhibit CoV values of 0.134 and 0.129 and MAPE% values around 11.69 and 11.00. These results clearly demonstrate that the proposed design, utilizing symbolic regression, greatly improves accuracy by significantly reducing error indices compared to conventional standards, making it a more reliable method for predicting column strength under eccentric compression.

**Fig. 9:**
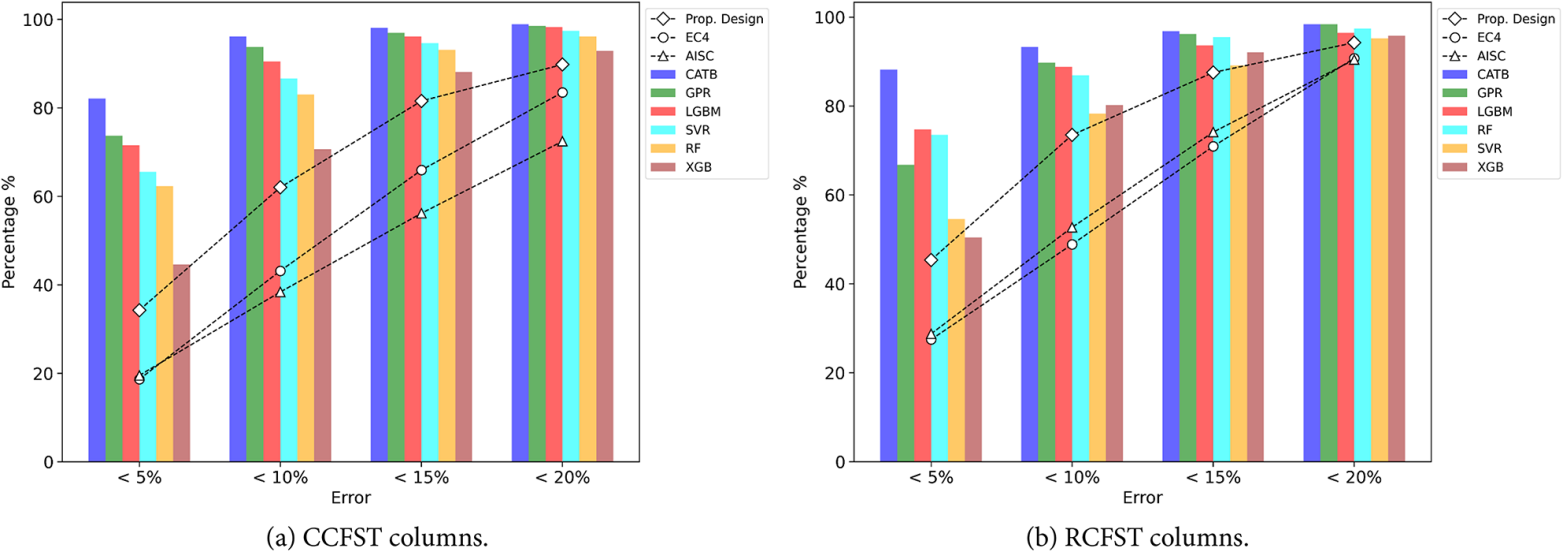
Prediction errors of previous models and established ML models.

Figure 9 demonstrates that most of the introduced ML models outperform traditional design standards in terms of predictive accuracy, particularly within the 10% error range. For CCFST columns, the CATB, GPR, and LGBM models capture over 90% of experimental samples within this error range, specifically 96.1%, 93.8%, and 90.5%, respectively. In contrast, the proposed design, EC4, and AISC360 standards capture 62.0%, 43.2%, and 38.4% of samples within the same error margin. This highlights the superior performance of the ML models in predicting the eccentric capacity of CCFST columns. Similarly, for RCFST columns, the CATB, GPR, LGBM, and RF models exhibit remarkable accuracy, with over 86% of samples falling within the 10% error range. Specifically, CATB achieves 93.3%, GPR 89.8%, LGBM 88.8%, and RF 86.9%. In comparison, the proposed design captures 73.5% of samples, whereas EC4 and AISC360 capture 48.9% and 52.7%, respectively. These results confirm that the ML models, particularly CATB, GPR, and LGBM, provide significantly more accurate predictions than the traditional design standards. Furthermore, the proposed design also shows better performance than EC4 and AISC360, capturing nearly 1.5 times more samples within the 5% and 10% error ranges. While the ML models excel in precision, the proposed design still delivers acceptable results, offering a more practical solution compared to the black-box nature of many ML models.

**Fig. 10:**
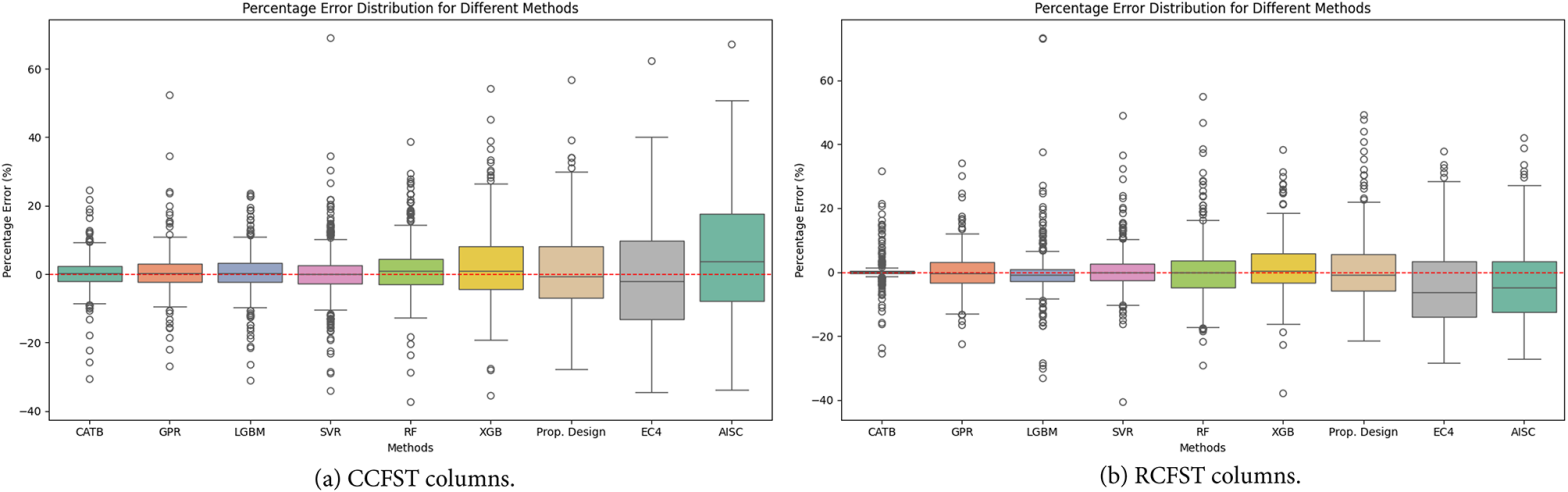
Box plot of prediction errors of previous models and established ML models.

A box plot^41,44^, also known as a box-and-whisker plot, visually represents the distribution of a dataset, summarizing its central tendency and variability. It consists of a rectangular box and whiskers extending from both ends. The box represents the interquartile range (IQR), which encompasses the middle 50% of the data, defined by the lower quartile (Q1) and upper quartile (Q3). A line within the box indicates the median, while the whiskers extend to the minimum and maximum values within 1.5 times the IQR. As shown in Fig. 10, the box plot is used to compare the percentage error distributions across various previous models and established ML models. For the CATB model, the box plot reveals that both the mean and median values are very close to zero, as shown in Fig. 10. Furthermore, the lower quartile (Q1), upper quartile (Q3), and whiskers of the CATB model demonstrate a smaller percentage error range compared to other ML models. These results highlight the CATB model as a reliable choice for predicting the buckling coefficient. In addition, the proposed design, utilizing symbolic regression, introduces better accuracy compared to conventional standards, making it a more reliable method for predicting column strength under eccentric compression.

## Limitations and future research directions

This section discusses the limitations of the developed data-driven models and identifies potential directions for future research. The proposed model’s validity is limited to the range of input parameter values specified in Table 2, which outlines the model’s applicability and defines the boundaries within which reliable predictions can be achieved. While the C-SR model demonstrates significant advantages, there are some limitations to consider. The model’s accuracy may decrease for extreme input values or novel column configurations outside the range of the training data. Future studies could investigate methods to enhance its generalization capability for such scenarios. Additionally, the current study focuses on columns under eccentric loading in one direction. Extending the analysis to include other loading conditions, such as eccentricity in the short direction or combined loading scenarios, could provide a more comprehensive understanding of CFST column behavior. Furthermore, exploring the application of the C-SR model to other types of composite columns, such as double-skin CFST or hybrid composite columns, could broaden its applicability. In summary, integrating ML-based approaches with physical models presents a promising tool for accurately predicting the eccentric strength of CCFST and RCFST columns, providing valuable insights for engineering applications. Future work will aim to address these limitations and explore additional avenues for enhancing the model’s versatility and robustness.

## Conclusions

In conclusion, this study compiled a comprehensive database of 464 experimental tests for the eccentric strength of CCFST columns and 313 RCFST column tests from various research papers. It employed symbolic regression (SR) techniques to refine and calibrate the design code provisions. From the evaluation results, the following conclusions can be drawn:

- The integration of symbolic regression with design code provisions not only improves the accuracy of eccentric strength predictions for CFST columns but also preserves the interpretability and alignment of the developed models with existing code standards.
- The proposed code-based symbolic regression (C-SR) model effectively combines the advantages of mechanical and black-box approaches, introducing strong performance with µ values of 1.006 and 0.997 and CoV values of 0.117 and 0.098 for CCFST and RCFST columns, respectively. These results demonstrate the model’s acceptable predictive accuracy and robustness.
- Compared to design standard codes, including EC4^26^ and AISC360^27^, the C-SR model shows better predictive performance, with improved CoV values and concentrated prediction-to-test ratios around unity.
- The machine learning models (CatBoost, GPR, LightGBM) applied in this study exhibit excellent predictive accuracy for both CCFST and RCFST columns, as evidenced by strong correlations between experimental and predicted values and minimal prediction errors (low MAPE% and RMSE values).
- While the CATBoost model demonstrates superior performance with CoV values of 0.052 for CCFST and 0.048 for RCFST columns, its black-box nature limits practical application in engineering design, highlighting the need for more interpretable models like the C-SR model.
- Although this study focuses on eccentric strength predictions, future research could extend the C-SR model to other loading conditions, such as eccentricity in the short direction, and explore its application to different types of composite columns.

The C-SR model not only enhances the prediction accuracy of the eccentric strength of CFST columns but also effectively combines the advantages of mechanical and black-box approaches. Unlike purely data-driven methods, the C-SR model offers explicit mathematical equations, making it more accessible and reliable for engineers in practical applications. In summary, integrating the ML-based approach with physical models presents a promising tool for accurately predicting the eccentric strength of CCFST and RCFST columns, providing valuable insights for engineering applications.

## Electronic supplementary material

Below is the link to the electronic supplementary material.

Supplementary Material 1

Supplementary Material 2

Supplementary Material 3

Supplementary Material 4

Supplementary Material 5

Supplementary Material 6

Supplementary Material 7

Supplementary Material 8

Supplementary Material 9

Supplementary Material 10

Supplementary Material 11

Supplementary Material 12

Supplementary Material 13

Supplementary Material 14

Supplementary Material 15

Supplementary Material 16

Supplementary Material 17

Supplementary Material 18

Supplementary Material 19

Supplementary Material 20

Supplementary Material 21

Supplementary Material 22

Supplementary Material 23

Supplementary Material 24

Supplementary Material 25

Supplementary Material 26

Supplementary Material 27

Supplementary Material 28

Supplementary Material 29

## Data Availability

All data generated or analyzed during this study are included in this published article and available in a public repository: https://github.com/kmegahed/eccentric-capacity-of-CFST-columns.
