# Supplementary material for: Symbolic regression for strength prediction of eccentrically loaded concrete-filled steel tubular columns
Source: Sci Rep. 2025 Jan 24;15:3085. doi: 10.1038/s41598-025-85371-x (PMC11761456; doi:10.1038/s41598-025-85371-x)
Supplement: Supplementary file 1 — Supplementary Material 1 [file 41598_2025_85371_MOESM1_ESM.docx]

**Derivation of eccentric capacity of CCFST sections (P-M interaction diagram)**


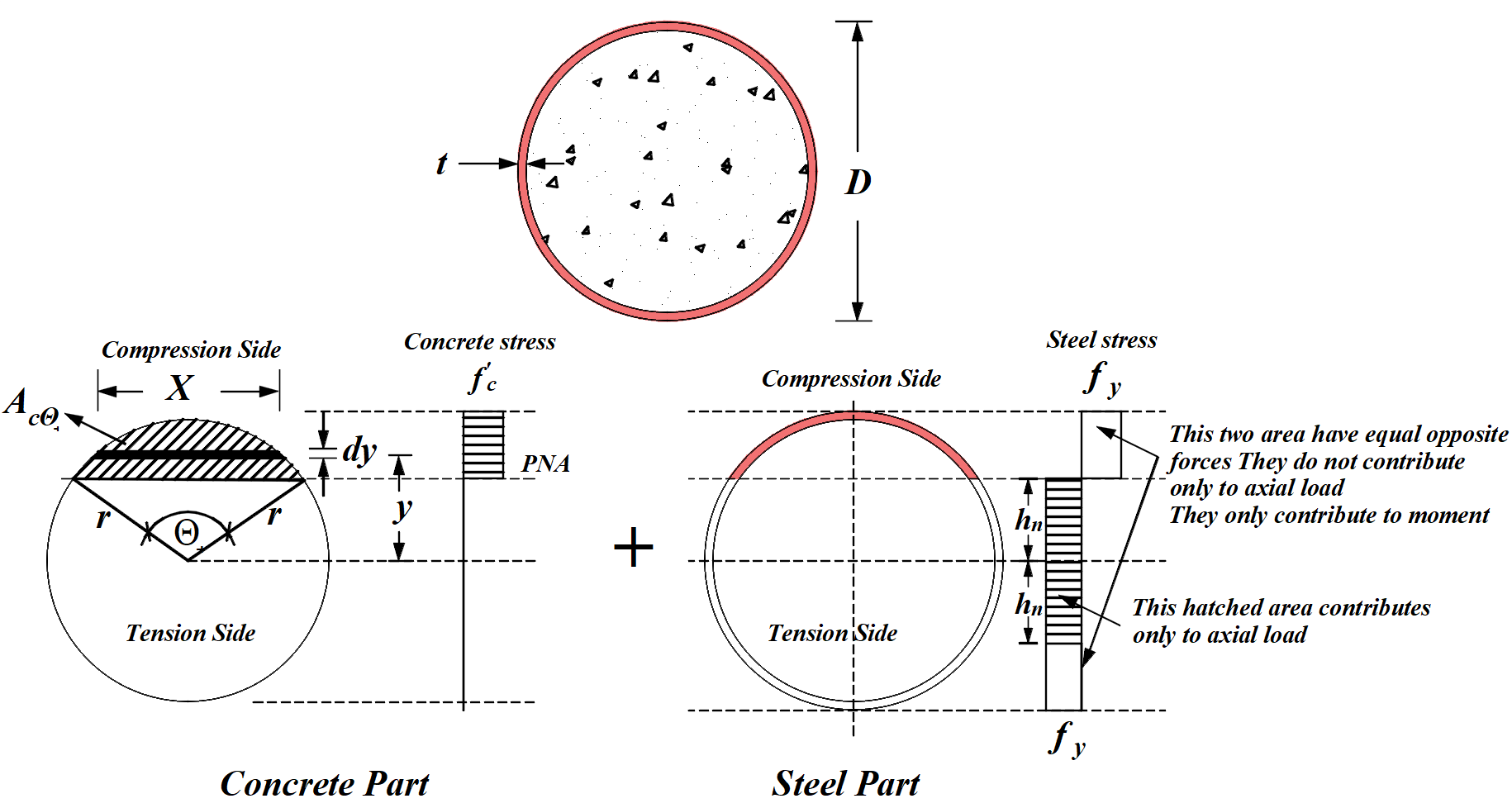


**For concrete part**

Area of compression concrete is $A_{c\theta}=A_{c}\frac{\left( \theta-\sin\theta\right)}{2\pi}$, where $A_{c}=\pi r^{2}=\frac{\pi\left( D-2t \right)^{2}}{4}$

Axial resistance = $A_{c\theta}f_{c}^{'}=f_{c}^{'}A_{c}\frac{\left( \theta-\sin\theta\right)}{2\pi}$

Bending moment resistance around column center point =$\int f_{c}^{'}y dA$

where $dA=Xdy=2\sqrt{r_{2}^{2}-y^{2}}dy$

for $y=r\sin\left( \frac{\theta}{2} \right), X=2r\cos\left( \frac{\theta}{2} \right)$ then $dy=\frac{r}{2}\cos\left( \frac{\theta}{2} \right)d\theta$

Then, bending moment resistance =$\int_{0}^{\theta} f_{c}^{'}r^{3}\sin^{2} \left( \frac{\theta}{2} \right)\cos\left( \frac{\theta}{2} \right)d\theta=\frac{2}{3}f_{c}^{'}r^{3}\sin^{3} \left( \frac{\theta}{2} \right)=0.5Z_{c}f_{c}^{'}\sin^{3} \left( \frac{\theta}{2} \right)$ where $Z_{c}=\frac{4}{3}\pi r^{3}=\frac{\pi\left( D-2t \right)^{3}}{6}$

**For steel part**

The hatched rea of steel (the net area contributing to tension) is $=A_{s}\left[ 1-\frac{\left( \theta-\sin\theta\right)}{\pi} \right]$, where $A_{s}=\frac{\pi D^{2}}{4}-\frac{\pi\left( D-2t \right)^{2}}{4}$

Axial resistance = $Af_{y}=A_{s}f_{y}\left[ 1-\frac{\left( \theta-\sin\theta\right)}{\pi} \right]$

Bending moment resistance around column center point can be computed similar to concrete part by considering subtracting two circles effect, the first one has D diameter, and the second one has (D-2t) diameter then multiplying twice due to the tension side also contribute the moment while neglecting tension for concrete as shown in above figure.

The bending moment resistance $=Z_{s}f_{y}\sin^{3} \left( \frac{\theta}{2} \right)$ where $Z_{s}=\frac{\pi D^{4}}{4}-\frac{\pi\left( D-2t \right)^{4}}{4}$

**Calculating angle** $\boldsymbol{\theta}$

By equilibrium, sum of axial resistance of steel and concrete parts mentioned above are:

$$P_{u}=sum of axial forces=f_{c}^{'}A_{c}\frac{\left( \theta-\sin\theta\right)}{2\pi}-A_{s}f_{y}\left[ 1-\frac{\left( \theta-\sin\theta\right)}{\pi} \right]$$

Then

$\theta-\sin\theta=\rho\pi$ with $\rho=\frac{P_{u}+P_{s}}{0.5P_{c}+P_{s}}, P_{c}=A_{c}f_{c}^{'}, P_{s}=A_{s}f_{y}$

**Column bending capacity**

Summing the bending moment resistance of concrete and steel pars, we get:

$$M_{n}=Z_{s}f_{y}\sin^{3} \left( \frac{\theta}{2} \right)+0.5Z_{c}f_{c}^{'}\sin^{3} \left( \frac{\theta}{2} \right)=\left( M_{s}+0.5M_{c} \right)\sin^{3} \left( \frac{\theta}{2} \right)$$

with $M_{s}=Z_{s}f_{y}, M_{c}=Z_{c}f_{c}^{'}$
