# Supplementary figures and images for: Symbolic regression for strength prediction of eccentrically loaded concrete-filled steel tubular columns
Source: Sci Rep. 2025 Jan 24;15:3085. doi: 10.1038/s41598-025-85371-x (PMC11761456; doi:10.1038/s41598-025-85371-x)

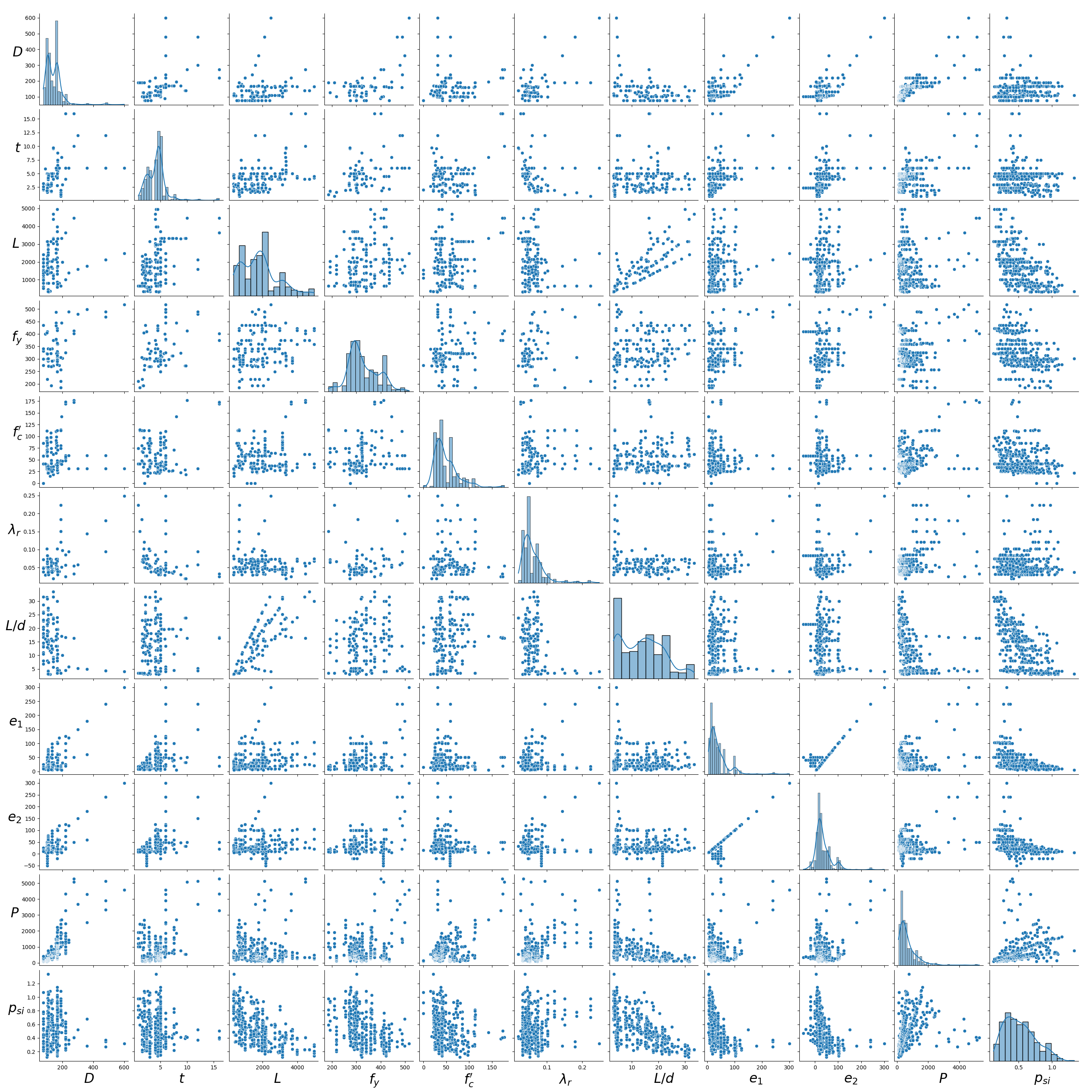

Supplement: Supplementary file 6 — Supplementary Material 6 [file 41598_2025_85371_MOESM6_ESM.png]

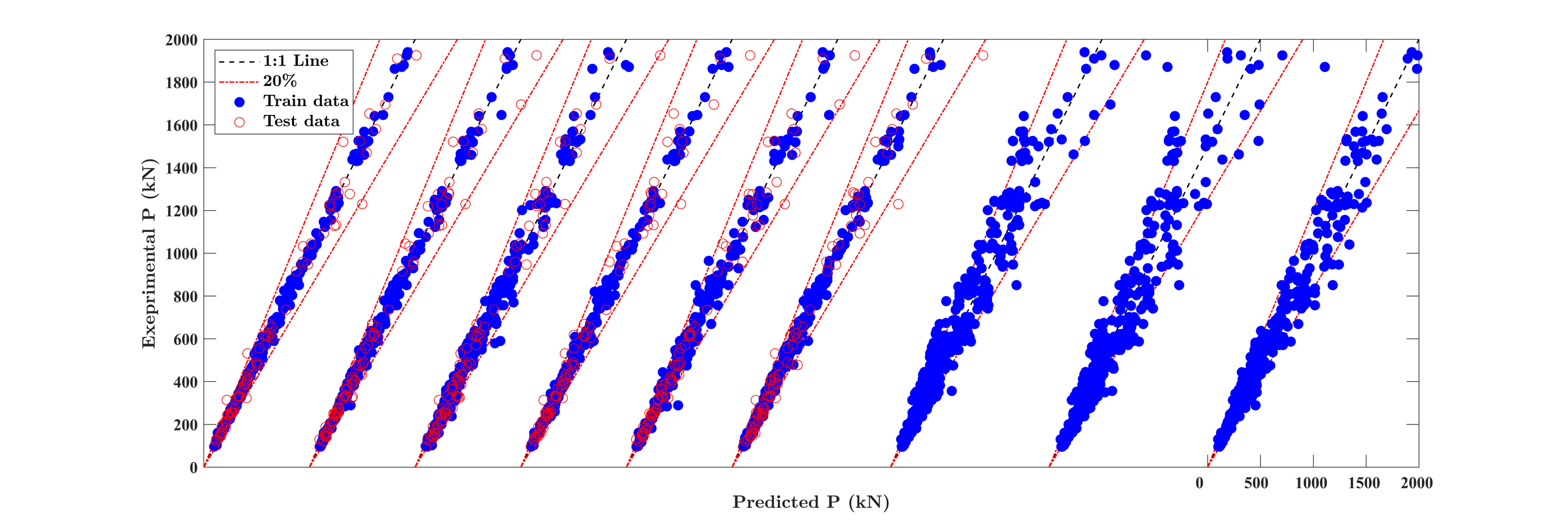

Supplement: Supplementary file 13 — Supplementary Material 13 [file 41598_2025_85371_MOESM13_ESM.png]

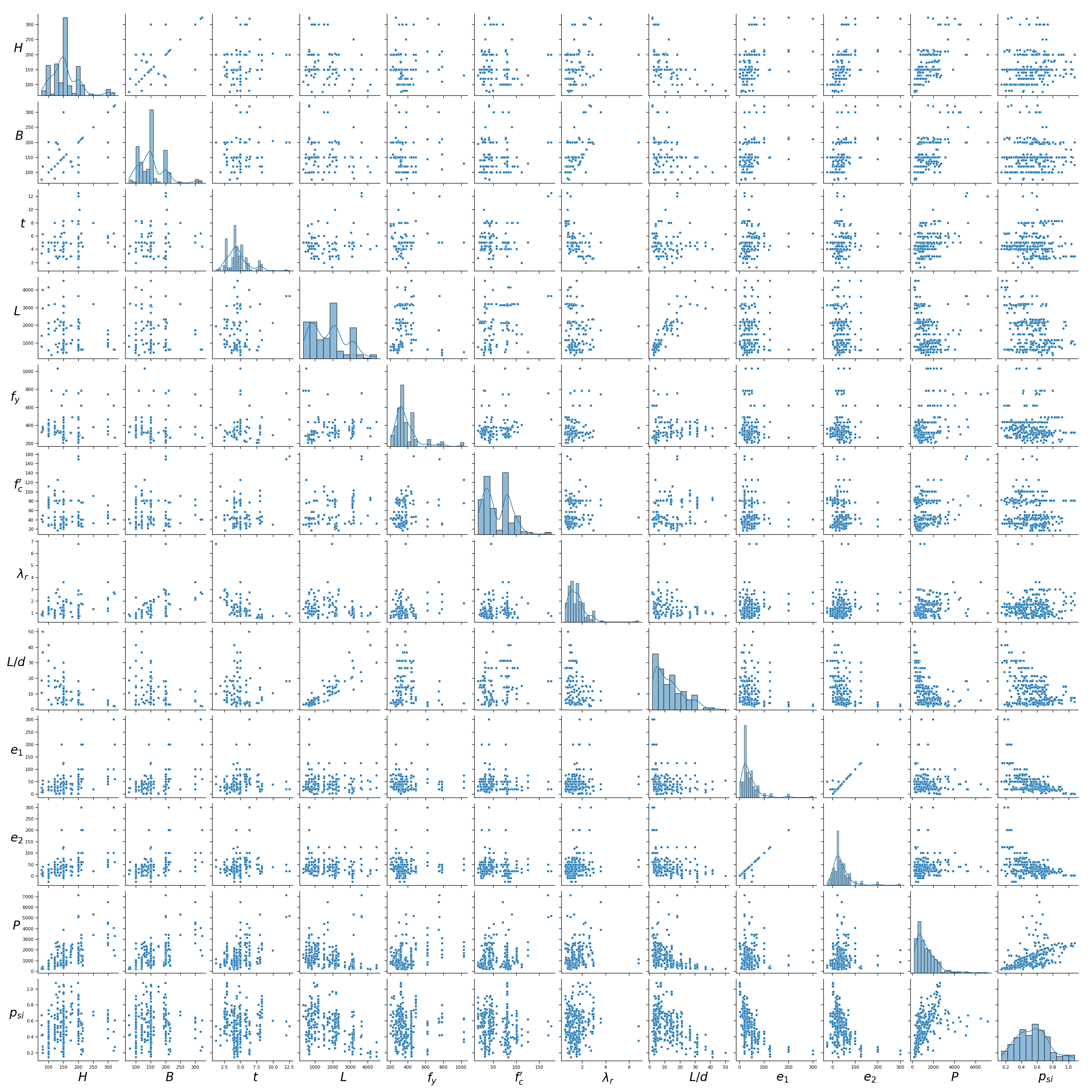

Supplement: Supplementary file 17 — Supplementary Material 17 [file 41598_2025_85371_MOESM17_ESM.png]

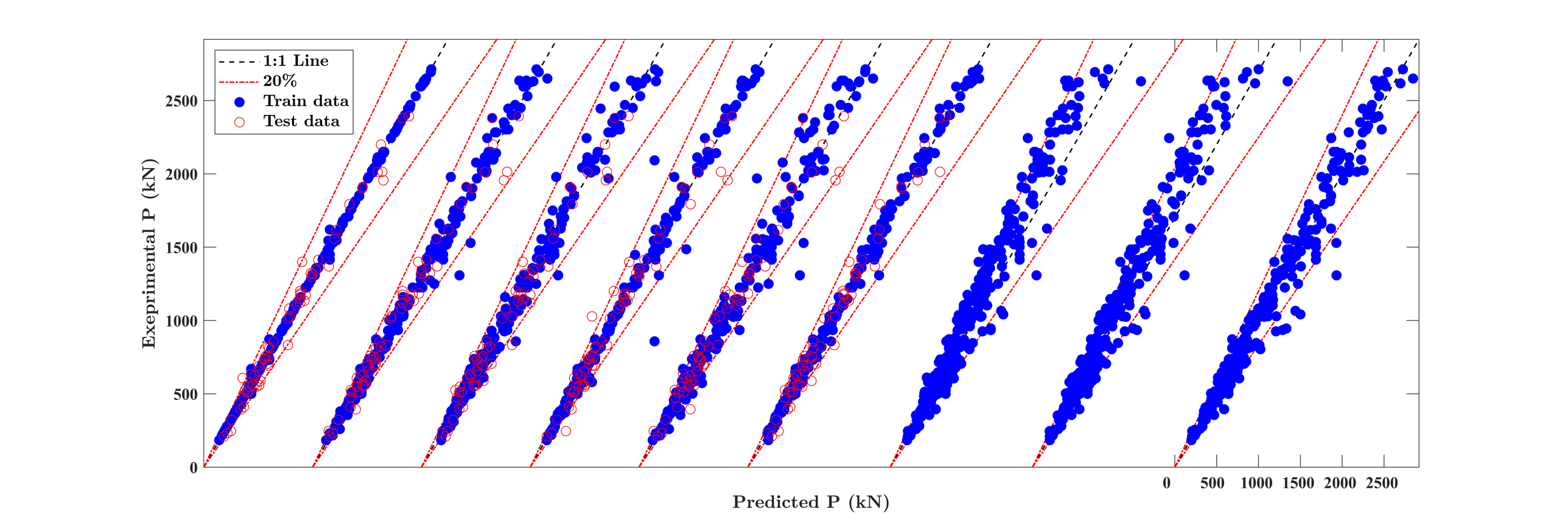

Supplement: Supplementary file 27 — Supplementary Material 27 [file 41598_2025_85371_MOESM27_ESM.png]
